# Alpha-B-Crystallin overexpression is sufficient to promote tumorigenesis and metastasis in mice

**DOI:** 10.1186/s40164-022-00365-z

**Published:** 2023-01-09

**Authors:** Behnam Rashidieh, Amanda Louise Bain, Simon Manuel Tria, Sowmya Sharma, Cameron Allan Stewart, Jacinta Ley Simmons, Pirjo M. Apaja, Pascal H. G. Duijf, John Finnie, Kum Kum Khanna

**Affiliations:** 1 QIMR Berghofer Medical Research Institute, 300 Herston Road, Herston, Brisbane, QLD 4006 Australia; 2 School of Biomedical Sciences, Faculty of Medicine, University of Queensland, Brisbane, QLD 4006 Australia; 3 School of Environment and Science, Griffith University, 170 Kessels Road, Nathan, Brisbane, QLD 4111 Australia; 4 South Australian Health and Medical Research Institute, Lifelong Health, Organelle Proteostasis Diseases, Adelaide, SA 5000 Australia; 5 Department of Molecular and Biomedical Sciences, University of Adelaide, Adelaide, SA 5000 Australia; 6 College of Public Health and Medicine, Flinders University, Bedford Park, SA 5042 Australia; 7 School of Biomedical Sciences, Faculty of Health, Queensland University of Technology (QUT), Brisbane, QLD 4000 Australia; 8 Centre for Data Science, Queensland University of Technology (QUT), Brisbane, QLD 4000 Australia; 9 Centre for Genomics and Personalised Health, Queensland University of Technology, Brisbane, QLD 4000 Australia; 10 Cancer and Aging Research Program, Queensland University of Technology, Brisbane, QLD 4000 Australia; 11 Institute of Clinical Medicine, Faculty of Medicine, University of Oslo, 0372 Oslo, Norway; 12 Department of Medical Genetics, Oslo University Hospital, 0450 Oslo, Norway; 13 Discipline of Anatomy and Pathology, Adelaide Medical School, University of Adelaide, Adelaide, SA 5000 Australia

**Keywords:** *Cryab* mouse model, Tumorigenesis, Metastasis, Angiogenesis, Heat-shock protein

## Abstract

**Background:**

αB-Crystallin is a heat shock chaperone protein which binds to misfolded proteins to prevent their aggregation. It is overexpressed in a wide-variety of cancers. Previous studies using human cancer cell lines and human xenograft models have suggested potential tumor promoter (oncogene) roles for αB-Crystallin in a wide-spectrum of cancers.

**Methods:**

To determine the causal relationship between *CRYAB* overexpression and cancer, we generated a *Cryab* overexpression knock-in mouse model and monitor them for development of spontaneous and carcinogen (DMBA)-induced tumorigenesis. In order to investigate the mechanism of malignancies observed in this model multiple techniques were used such as immunohistochemical characterizations of tumors, bioinformatics analysis of publically available human tumor datasets, and generation of mouse embryonic fibroblasts (MEFs) for in vitro assays (clonogenic survival and migration assays and proteome analysis by mass-spectrometry).

**Results:**

This model revealed that constitutive overexpression of *Cryab* results in the formation of a variety of lethal spontaneous primary and metastatic tumors in mice. In vivo*,* the overexpression of *Cryab* correlated with the upregulation of epithelial-to-mesenchymal (EMT) markers, angiogenesis and some oncogenic proteins including Basigin. In vitro, using E1A/Ras transformed MEFs, we observed that the overexpression of *Cryab* led to the promotion of cell survival via upregulation of Akt signaling and downregulation of pro-apoptotic pathway mediator JNK, with subsequent attenuation of apoptosis as assessed by cleaved caspase-3 and Annexin V staining.

**Conclusions:**

Overall, through the generation and characterization of *Cryab* overexpression model, we provide evidence supporting the role of αB-Crystallin as an oncogene, where its upregulation is sufficient to induce tumors, promote cell survival and inhibit apoptosis.

**Supplementary Information:**

The online version contains supplementary material available at 10.1186/s40164-022-00365-z.

## Background

Cancer is a complex genetic disease that stems from the mutations of various genes. These mutations lead to the hallmarks of cancer which favor survival, angiogenesis, invasion, and metastasis [1]. However, the mechanisms that create these advantages also lead to challenges such as proteostasis stress, oxidative stress and hypoxia that can stimulate cell death [2, 3]. Cancer cells must overcome these stress responses to develop from a benign tumor into invasive metastatic cancer through rewiring of proteostatic processes such as upregulation of protein folding chaperones including the αB-Crystallin protein encoded by the CRYAB gene to avoid proteotoxicity [2]. Through oligomerizing with other heat-shock proteins, αB-Crystallin allows misfolded or unfolded proteins to be sequestered and prevents their detrimental aggregation which would otherwise create a harmful environment.

The expression of *CRYAB* has been thoroughly investigated in the context of a wide range of cancers where it has been validated as a prognostic marker [4]. In clear cell and papillary type renal cell carcinoma and colorectal cancer, *CRYAB* is used as a marker for higher tumor stage and distant metastases, and in osteosarcoma, it is a marker for increased metastases and poor chemotherapy response [4]. In breast cancer, *CRYAB* is a marker for aggressive behavior in triple-negative basal-like breast cancer and mammary metaplastic carcinoma and its overexpression is associated with the presence of lymph nodal and brain metastasis and relapse [5]. *CRYAB* is also categorized as a marker for lower overall survival in cancers such as renal cell carcinoma, osteosarcoma, colorectal, hepatocellular carcinoma, gastric, ovarian, and non-small cell lung cancer [4].

At the molecular level, *CRYAB* has been shown to disrupt apoptosis through both the intrinsic and extrinsic pathways [2] via inhibition of pro-apoptotic Bcl-2 family proteins including Bax and Bcl-xs which contribute to caspase 3 activation. In human mammary epithelial cells, the overexpression of *CRYAB* led to disruption of normal mammary acinar morphology and induction of neoplastic changes [4]. These phenotypes resulted from the activation of survival pathways such as p38, AKT and ERK with *CRYAB* overexpression which could in part be rescued through inhibition of the MEK/ERK pathway [4]. Furthermore, breast cancer cells can induce VEGF expression in co-cultured endothelial cells via activation of the unfolded protein response and its downstream effector *CRYAB* which protects VEGF from proteolytic degradation [6].

Despite multiple studies demonstrating a role for *CRYAB* in promoting tumorigenesis in vitro, it remains undetermined whether there is a causal link between *CRYAB* overexpression and cancer formation. Here, we demonstrate for the first time, using a transgenic mouse model, that overexpression of *Cryab* is sufficient for de novo tumorigenesis. *Cryab* -overexpressing mice show high incidence (almost 50%) of spontaneous tumor formation with a wide-spectrum of highly proliferative primary and metastatic tumors. Notably, *Cryab* overexpression significantly increased tumor load in a carcinogen-induced tumor model. Using the mouse embryonic fibroblasts (MEFs) from this mouse model, we show that *Cryab* overexpression alters multiple signaling pathways, particularly those related to apoptosis, survival, and metastasis which have potential implications for tumor initiation and therapy development.

## Materials and methods

### Generation of the targeting construct

Rosa26-UbiC- Cryab floxed mice were generated by Ozgene (Perth, WA, Australia). To generate the *Cryab* knock-in model, we designed a targeting vector containing a Flag-tagged *Cryab* cDNA (fl-Tg) preceded by a human ubiquitin C (UbiC) promoter as well as lox-P flanked polyadenylation (pA +) stop region, with a downstream flippase recombinase target site-flanked neomycin resistance cassette (PGK-NEO) for embryonic stem cell (ESC) selection. Genomic targeting of the construct was attained in ESCs of wild-type BALB/C, by utilizing standard homologous recombination and blastocyst manipulation techniques. Gene manipulation was validated by Ozgene using Southern blot analysis, with probes against both the endogenous coding region and NEO selection cassette following restriction digest of genomic DNA with the EcoRV restriction enzyme.

### Generation of the ubiquitous Cryab knock-in mouse model

*Cryab* knock-in mice were generated by crossing heterozygous (het) Rosa26Ubiq-polyA-flTg(Neo)/wt mice with FLPe mice to remove the PGK-Neo cassette, followed by backcrossing to C57BL/6 wild-type mice to excise the FLPe transgene. Rosa26UbiC-polyA-flTg(Neo)/Wt mice were then crossed with Rosa26EIIA-Cre mice (from Ozgene) to remove the (pA +) stop region, allowing overexpression of *Cryab* cDNA from the Rosa26 locus (Rosa26Tg/Tg). These mice were then crossed to BALB/C Wt mice to separate the Rosa26EIIA-Cre and RosT26Tg/Tg alleles. The resulting *Cryab* heterozygous (*Cryab*^Wt/Tg^) mice were intercrossed to generate 3 genotypes: Wt (*Cryab*^Wt/Wt^), het (*Cryab*^Wt/Tg^), and homozygous (*Cryab*^Tg/Tg^) mice. Wt control mice used for Kaplan–Meier survival analysis served as joint controls with [7].

### Animal husbandry

All experimental animals were maintained on a mixed background (Balb/c X C57BL/6) strain. Mice were housed at 25 ℃ in a 12 h light–dark cycle.

### Cell culture

Mouse embryonic fibroblasts (MEFs) were generated as described previously [8]. Briefly, primary MEFs were immortalized and transformed by E1A/Ras oncogene. All cell lines were annually authenticated using short tandem repeat (STR) profiling and routinely tested for Mycoplasma infection by scientific services at QIMR Berghofer Medical Research Institute.

The cells were maintained in culture in Dulbecco’s Modified Eagle’s Medium (DMEM) (Life Technologies TM, Carlsbad, CA, USA) containing 20% Fetal Bovine Serum (SAFC BiosciencesTM, Lenexa, USA) 1% penicillin–streptomycin (Life Technology TM) and 1% Amphotericin B.

### siRNA transfection

MEFs were plated in 6 well plates at density of 200,000 cells/well followed by double reverse transfection using 20 nM of siRNAs with following sequence (siRNA against BSG):

UTR 5' CCUUCUGAAGUGUUGUCACUACAGC 3'

5' GCUGUAGUGACAACACUUCAGAAGGGA 3'

Exon2  5' GGUUUGAAGGGAAUGCUCCAAACGA 3'.

5' UCGUUUGGAGCAUUCCCUUCAAACCAC 3'

Exon6 5' GUCACAGCUGACCAUCAGCAACCTT 3'.

5' AAGGUUGCUGAUGGUCAGCUGUGACUU 3'

Scr 5’ CAAUGUUGAUUUGGUGUCUGCA 3’.

5’ UGAAU AGGAUUGUAAC 3’

### Cell proliferation assay

Cell proliferation was performed as previously described [9]. Cells were plated in a 24-well plate at density of 5000 cells per well in duplicate and cultured overnight. The following morning, plates were transferred to an incubator equipped with an IncuCyte^®^ S3 Live-Cell Analysis system (Essen BioSciences Inc, USA) for 6 days. Cell confluency was analyzed using the in-built IncuCyte^®^ S3 software.

### Clonogenic assays

Cells were plated on a 6-well plate at a density of 500 cells per well and incubated for two weeks to determine colony viability. Then, colonies were fixed with 0.05% crystal violet for 30 min, washed and quantified for crystal violet colony counting and measurement by imaging on a GE InCell 2000 microscope and analysis by GE InCell 2000 3-D Deconvolution Software (GE Health care, Life Sciences, USA) and total surface area quantified by ImageJ v1.53q.

### Transwell assay

To measure cell migration 10^4^ cells were seeded 0.05% FBS onto the top of a transwell insert with 8 µM pores (Corning Inc. New York, USA) placed into a 24-well cell culture dish where 20% FBS was used as a chemoattractant in the base of the culture dish.Cells were incubated in the transwell plate at 37 ℃ for 20 h and the migrated cells on the lower surface fixed at − 20 ℃ in ice cold MeOH for 30 min. After fixing, cells were stained with Crystal violet (0.5% (w/v) in 25% MeOH (v/v)) for 30 min. To quantitate cell migration 6 fields of view per membrane were photographed with EVOS^™^ FL Auto 2 Imaging System (Thermo Scientific™ Invitrogen™), followed by quantification by ImageJ v1.53q (National Institutes of Health, USA).

### Apoptosis detection assay

For detection of apoptotic and necrotic cells, we used Annexin V and propidium iodide (PI) staining. Cells were plated at a density of 1 × 10^5^ in triplicate in 6-well cell culture plates and incubated for 16 h. Cells were harvested with trypsin–EDTA and washed twice in media containing FBS. Cell pellets were resuspended in 100 µL of 1X binding buffer (0.1 M HEPES/NaOH (pH 7.4), 1.4 M NaCl, 25 mM CaCl2) and 2 µL of Fluorochrome conjugated Annexin V-FITC (Invitrogen^™^, USA). The cells were incubated for 15–20 min on ice followed by addition of 5 µL of PI (1 mg/mL) and 400 µL of binding buffer. Annexin V-FITC binding was detected via flow cytometry (Ex = 488 nm; Em = 350 nm) using FITC signal detector. The analysis was performed using FlowJo v. 10.0.6 (Tree Star, Ashland, Oregon, USA).

### Immunoblotting

Cells were prepared for lysis as described previously [8] with indicated antibodies (Table 1). The Super Signal chemiluminescent ECL‐plus (Amersham) was used for signal detection.

**Table 1:** Primary and secondary antibodies used for Western blot

Antibody	Company	Cat. No	Dilution
Alpha B Crystallin	Novus	NBP2-49,246	1:500
Basigin (CD147)	Abcam	ab230921	1:1000
GAPDH	R&D Systems	RDS2275PC100	1:2000
Vinculin	Cell signalling technology	13,901	1:2000
β-Catenin	Cell signalling technology	9582	1:1000
Cleaved Caspase-3	Cell signalling technology	9664	1:500
pAKT^S473^	Cell signalling technology	4060	1:1000
AKT	Cell signalling technology	9271	1:1000
p-JNK	Cell signalling technology	4671	1:1000
Rabbit secondary (peroxidase)	Sigma Aldrich	A0545	1:5000
Mouse secondary (peroxidase)	Sigma Aldrich	A9044	1:5000

### Polymerase chain reaction

PCR was performed as described previously [8] using Genomic DNA from animal tissue or cell pellet was extracted using QuickExtract DNA solution (Gene target solutions, QE09050).

### Mass-spectrometry

*Cryab*^Wt^ and *Cryab*^Tg^ MEFs were treated for 1 h at 43ºC to generate heat-shock stress before processing samples in triplicates for in-situ protein digestion as per previously described method [10]. For mass-spectrometry samples were loaded on to a Waters M-Class SYM100 trap column (180 um × 20 mm ID) for 6 min at a flow rate of 5 µl/min with 95% Solvent A (0.1% FA in water), and subsequently separated on a Waters BEH130 analytical column (75 µm × 200 mm ID). Columns were equipped on a Waters nanoACQUITY UPLC coupled with a Thermo Orbitrap Fusion mass spectrometer. The solvent gradient ran at 300 nl/min and started at 92% Solvent A before ramping up to 27% Solvent B (0.1% FA in acetonitrile) over 45 min. This was followed by column washing and reequilibration for a total run time of 60 min. MS spectra were acquired in the mass range = 350–1800 m/z (orbitrap resolution = 60,000). Fragmentation for MS/MS spectra were acquired in the orbitrap at a resolution of 15,000 with a collision energy 30. The AGC target was 5e4, with a maximum ion injection time of 40 ms. The isolation window was set to 1.2 m/z. Dynamic exclusion was set to 15 s and precursors with charge states from 2 to 6 were accepted for fragmentation.

Raw LCMS data was searched for protein IDs against the reviewed Uniprot mouse database (21,963 sequences, downloaded 24/04/2020) using Sequest HT on the Thermo Proteome Discoverer software (Version 2.2). Precursor and fragment mass tolerance were set to 20 ppm and 0.05 Da respectively. A maximum of two missed cleavages were allowed. A strict false discovery rate (FDR) of 1% was used to filter peptide spectrum matches (PSMs) and was calculated using a decoy search Carbamidomethylation of cysteines was set as a fixed modification, while oxidation of methionine and deamidation of glutamine and asparagine were set as dynamic modifications. Protein abundance was based on intensity of the parent ions and data was normalized based on total peptide amount.

Differentially expressed proteins were ranked based on upregulated ones over log_2_(0.6) or downregulated under log_2_(− 0.6) in *Cryab*^Tg^ MEFs against *Cryab*^Wt^ MEFs. The biological pathway enrichment analysis was performed using GO analysis, and MCL cluster annotations and visualized using Cytoscape [11] v.3.8.1

### DMBA tumorigenic treatments

DMBA (7,12-Dimethylbenz[a]anthracene) treatments consisted of a single administration of 50 μl of a solution 0.5% DMBA (Sigma) to the dorsal surface on postnatal day 5 of mice.

### Histopathological analysis and immunohistochemistry staining

Immunohistochemistry (IHC) was performed as previously described [8]. Briefly, for histopathologic investigation with hematoxylin and eosin (H&E), tissues were collected and fixed in formalin solution and embedded in paraffin blocks. Sections were prepared and stained following the standard procedure with indicated antibodies (Table 2) and imaged with Aperio AT turbo/FL scanner (Leica Biosystems, Buffalo Grove, IL, USA). Two independent pathologists then provided their comments, in a detailed report and highlighted the regions of interest for analysis. The H-Score value was calculated in a semi-quantitative fashion which incorporated both the intensity and the distribution of staining. This value was derived by summing the percent of positive staining cells at each intensity multiplied by the weighted intensity of staining for each tissue. The intensity evaluations were recorded as four intensity categories which were designated as 0 (no staining), 1 + (weak but detectable above control), 2 + (distinct), 3 + (strong). H-Scores were generated for the cohort of indicated number of regions of interest from tumor and adjacent tumor tissues. IHC image analysis and quantitation was performed using QuPath software version 0.2.3.  Correlation studies were performed in a blinded fashion for each target following by Pearson correlation coefficient (R) or linear regression as noted in figure's legend.

**Table 2:** Primary Antibodies used for IHC/IF

Antibody	Company	Cat. No
Alpha B Crystallin	Novus	NBP2-49,246
Basigin (CD147)	Abcam	ab230921
Vinculin	Cell signalling technology	13,901
β-Catenin	Cell signalling technology	9582
pAKT^S473^	Cell signalling technology	4060
E-cadherin	Dako	M3612
Vimentin	Cell signalling technology	5741
pan Cytokeratin	Dako	M351529
Smooth Muscle Actin	Biocare medical	CM001C
p53	Novocastra (Leica)	NCL-p53-CM5p
p44/42 MAPK (Erk1/2)	Cell signalling technology	4695
CD4	eBioscience	14–9766
CD8	eBioscience	14–0808
F4/80	Abcam	ab16911

### CRYAB expression analyses

In human tumors, *CRYAB* gene expression and gene expression signature analyses were performed in samples from The Cancer Genome Atlas (TCGA) as described in the Additional file 4: Additional Methods. All other CRYAB expression analyses were performed using samples from various datasets from the Gene Expression Omnibus (GEO, https://www.ncbi.nlm.nih.gov/geo) with the following accession numbers: normal cervix and early-stage cervical cancers (GSE7410), primary and metastatic renal cell carcinoma (GSE31232), primary tumors without and with metastasis in hepatocellular carcinoma (GSE45114), pancreatic cancer (GSE63124), breast cancer (GSE9893) and colorectal cancer (GSE87211), locally and distantly metastasized pancreatic cancer (GSE34153), prostate cancer (GSE74367) and lung cancer (GSE18549).

### Statistical analyses

All statistical analyses were performed utilizing GraphPad Prism v 9.0 software, using a general linear statistical model, as defined in each section. The error bar represents the mean ± standard deviation (SD) unless indicated otherwise. The statistical significance of the *p*-value is designated with an asterisk (*); *p*-values: * *p* < 0.05, ** *p* < 0.01, *** *p* < 0.001, and **** *p* < 0.0001.

## Results

### *CRYAB* is overexpressed and correlates with poor patient prognosis in multiple types of human cancers

To identify the pattern of *CRYAB* expression levels and copy number alterations in cancer, we performed pan-cancer analyses using TCGA datasets. A comparison of SNP array data revealed that *CRYAB* is gained or amplified in many cancers such as B-Cell neoplasms, non-small cell lung cancer, glioblastoma, melanoma, colorectal, bladder, pancreatic, breast, endometrial, renal, thyroid cancer, and sarcoma (Additional file 1: Fig. S1a). Furthermore, we found that there is a positive correlation between *CRYAB* copy number and expression level in multiple cancers including breast, ovarian, head and neck, colon and rectum, glioma and lung squamous cell carcinoma (Additional file 1: Fig. S1b). Additionally, comprehensive survival analyses showed significant associations between high *CRYAB* expression and poor overall patient survival in the vast majority of cancer types, particularly in gastric, bladder, lung cancers. (Additional file 1: Fig. S2).

Next, we investigated links between *CRYAB* expression and different features of cancers such as the dysregulation of signaling pathways, angiogenesis, increased proliferation and stemness, neoantigen production, immunoregulation and, tumor infiltration. Notably, this revealed the possible relationship between *CRYAB* expression and dysregulated signaling in different cancer types. For instance, in breast cancer (n = 753), there is a positive correlation between *CRYAB* expression and *Ras/MAPK* (r = 0.1863, p = 2.6 × 10^−7^); *PI3K/Akt* (r = 0.1904, p = 1.4 × 10^−7^); epithelial–mesenchymal transition (EMT) score (r = 0.2750, p = 1.6 × 10^−14^); apoptosis (r = 0.1238, p = 0.0007); autophagy (r = 0.1492, p = 6.3 × 10^−7^); angiogenesis ( r = 0.3059, p = 6.8 × 10^−25^); and hypoxia based on Elvidge et al. (r = 0.5320, p = 1.2 × 10^−79^) and negative correlation with hormone expression (Additional file 1: Fig. S3a, 3b and 3c). Consistent with this, *CRYAB* overexpression predominantly occurs in triple-negative breast cancer that lack expression of hormone receptors [5]*.* Notably, autophagy, angiogenesis and, hypoxia have a significant-positive correlation to *CRYAB* expression across several cancer types such as adenocarcinomas of colon, lung, prostate, and stomach (Additional file 1: Fig. S3c). In the clinical setting, autophagy and angiogenesis are linked to *CRYAB* overexpression and this gene is abundantly expressed in hypoxic regions of tumors; however, in cell-based in vitro assays the reoxygenation and subsequent generation of reactive oxygen species (ROS) activates *CRYAB* rather than hypoxia [2].

Next, to investigate correlation between gene expression data and estimation of the abundances of immune cell types, we conducted a Cibersort query which revealed a positive correlation between *CRYAB* expression and macrophage infiltration, particularly M2 macrophages, which play a significant immunosuppressive role in tumor microenvironment in bladder (r = 0.3954, p = 2.6e− 16, n = 397), colon (r = 0.4032, p = 1.1e− 18, n = 442), esophageal (r = 0.3692, p = 5.3e− 07, n = 174), head and neck squamous cell carcinoma (r = 0.1984, p = 6e− 06, n = 516), kidney (r = 0.2790, p = 2.2e− 06, n = 279), liver (r = 0.1832, p = 0.0005, n = 362), rectum (r = 0.5579, p = 3.8e− 14, n = 156) and, testicular germ cell tumors (r = 0.2269, p = 0.0054, n = 149). However, a negative correlation between *CRYAB* expression and macrophages was found in breast (r =− 0.1526, p = 4.2e− 07, n = 1090), prostate (r =− 0.1940, p = 8.4e− 05, n = 406) and sarcoma (r = 0.1945, p = 0.0035, n = 224) (Additional file 1: Fig. S3d). Additionally, we found negative correlation of *CRYAB* expression and lymphocytes in a large number of tumor types, suggesting reduced lymphocytic infiltration. Overall, the bioinformatic analysis suggests that *CRYAB* overexpression leads to poor outcome in several types of cancers and angiogenesis is significantly positively correlated to *CRYAB* expression in most cancers.

### *Cryab* overexpression in the mouse model causes spontaneous tumorigenesis in vivo

**Fig. 1:**
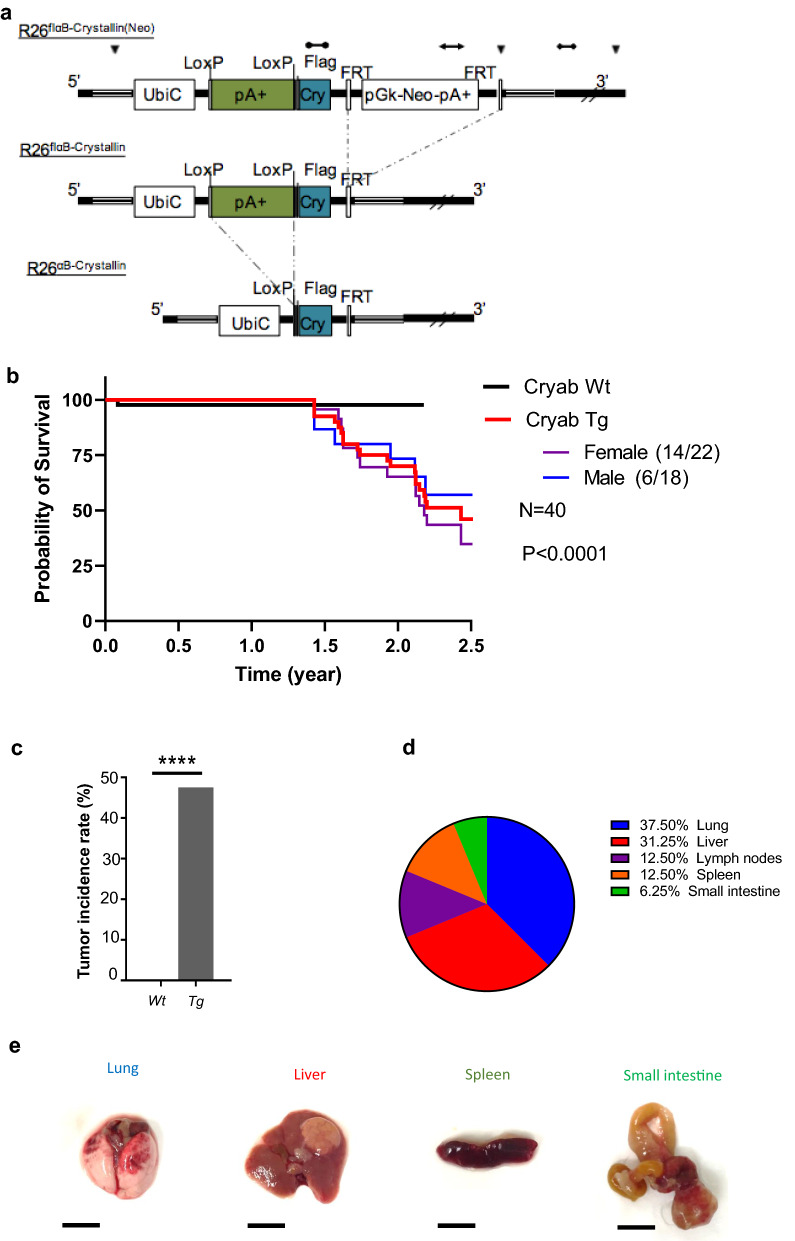
*Cryab* overexpression causes spontaneous tumorigenesis in vivo. **a** Schematic representation of αB-Crystallin overexpressing mice (Ozgene) developed by knocking in the transgene into the Rosa26 locus. The targeting vectors show recombination steps. Legend: FLPe recombination, Cre recombination, Homology arm, ▼ EcoRV site, transgene probe (tgP), 3’ probe (3’P), Neomycin probe (Neo). **b** Kaplan–Meier survival analysis of mice for indicated genotypes (n = 40 per group) Log-rank (Mantel–Cox) test was performed to determine P-value < 0.00001. **c** Percentage of cancer incidence rate among mice of indicated genotypes (n ≥ 40 per group); Fischer exact test was performed to determine P-value < 0.00001 (****). **d** The distribution of tumor spectrum in tissues of *Cryab* transgenic mice. **e** Representative images of gross morphology of tissues with different malignancies. (Scale bars, 1 cm)

Both mouse and human αB-Crystallin proteins are comprised of 175 amino acids and exhibit 98% sequence homology with just 4 amino acid mismatches [12]. Between these two species, the three sites of phosphorylation and the α-B crystallin domains are conserved (Additional file 1: Fig. S4a). Collectively, αB-Crystallin is a conserved protein in human and mouse. To assess whether in vivo overexpression *of Cryab* is sufficient to initiate tumor formation, we generated a Flag-*Cryab* knock-in mouse model to ubiquitously overexpress *Cryab* cDNA from the Rosa26 locus (Rosa26 Tg/Tg) (Fig. 1a). Correct targeting was validated by PCR on genomic DNA extracted from different genotypes (*Cryab*^Wt/Wt^ = Wt, *Cryab*^Wt/Tg^ = Het, *Cryab*^Tg/Tg^ = Tg or Hom) (Additional file 1: Fig. S4b) and protein expression across different organs of the mouse using immunoblotting analysis, respectively (Additional file 1: Fig. S4c). *Cryab*^Tg^ mice were viable and had no obvious developmental defects. Using this constitutive overexpression mouse model of *Cryab*, we monitored a cohort of wild type mice (hereafter referred to as *Cryab*^*Wt*^, n = 40) as a control group to compare with homozygous transgenic mice overexpressing αB-Crystallin (*Cryab*^*Tg*^, n = 40: 23 Females, 17 Males) over time for spontaneous tumor formation. Any mouse showing signs of distress and disease according to the ethical limitations was sacrificed for further histopathological investigation and considered as the experimental endpoint.

**Fig. 2:**
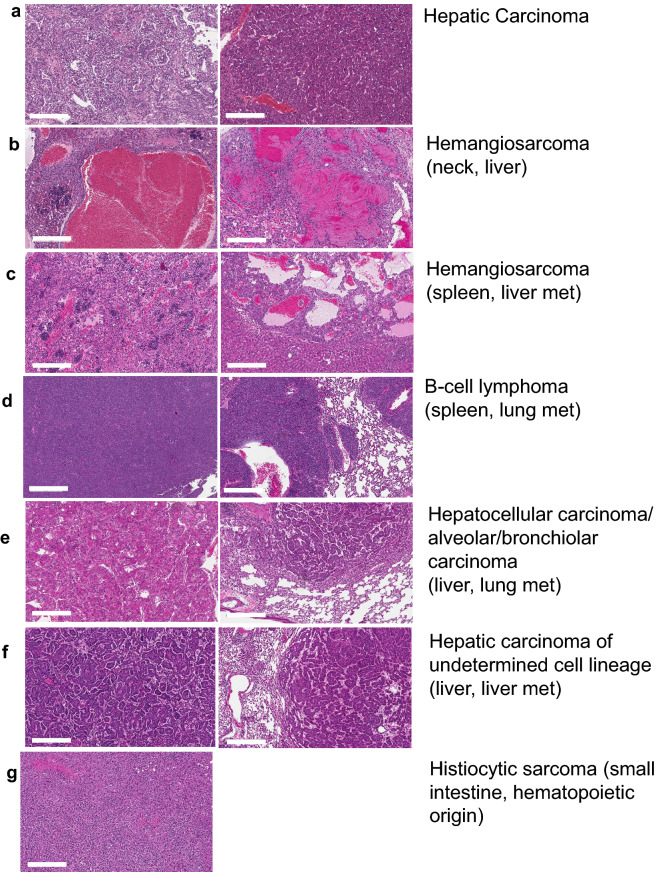
Characterization and classification of the primary tumors and metastasis. **a**–**g** Representative H&E stained microscopic images of selected sections of tumor-bearing *Cryab*^Tg^ mice as indicated in the figure, **a** hepatic carcinoma in liver; **b** Hemangiosarcoma in neck (left) and liver (right); Hemangiosarcoma in spleen (left) and liver metastasis (right); **d** B-cell lymphoma in spleen (left) and lung metastasis (right); **e** Hepatocellular carcinoma in liver (left) and alveolar/bronchiolar carcinoma in lung metastasis; **f** Hepatic carcinoma of undetermined cell lineage in liver (left) and liver metastasis; **g** Histiocytic sarcoma in the small intestine from hematopoietic origin. (scale bars, 300 µm)

*Cryab*^Tg^ mice (both males and females), were more susceptible to the formation of tumors compared to the control group as shown in Kaplan–Meier tumor-free survival analysis (Fig. 1b). The spontaneous tumorigenesis in *Cryab*^Tg^ started at 17 months and by 26 months half of these mice died due to tumor burden irrespective of gender differences, while in this time-frame just one mouse from the control group died due to an idiopathic cause. In terms of gender differences, we observed tumorigenesis in 33.33% of male (blue) and 63.63% of female (purple) *Cryab*^Tg^ mice. Therefore, murine females were more susceptible to the formation of tumors compared to males. Furthermore, all mice were subjected to examination for signs of macroscopic tumor formation across major organs and tissues including mammary glands, liver, lung, spleen, small intestine, kidney and lymphoid tissues. The major types of tumors observed originated from lung and liver followed by lymph nodes, spleen and small intestine (Fig. 1d, e, Table 3). The pathological investigation of tumorigenesis based on hematoxylin and eosin (H&E) staining revealed further details of the tumor spectrum including alveolar/bronchiolar carcinomas, hepatocellular carcinomas, B-cell lymphomas, sarcomas as well as liver and lung metastases (Fig. 2a–g, Table 3). Next, we examined αB-Crystallin expression in tumor and adjacent normal tissues by immunoblotting and we observed the higher expression level of αB-Crystallin in lung tumors compare to adjacent tissues as expected (Additional file 1: Fig. S4d). Collectively, these data highlight that *Cryab* overexpression is sufficient to cause a broad spectrum of spontaneous primary and metastatic tumors in mice with 50% incidence in the time-frame of this study.

**Table 3:** The distribution of cancer spectrum in Cryab transgenic mice

Malignancies	Incidence (frequency)	Features
Hemangiosarcoma	(30%)	This neoplasm was comprised of vascular spaces of varying size, sometimes containing thrombi, and lined by a pleomorphic population of proliferating endothelial cells with frequent mitotic figures present
Hepatic Carcinoma (hepatocellular carcinoma)	(25%)	There was trabecular growth in irregularly thick plates of neoplastic hepatocytes, which sometimes resembled normal hepatocytes, but often had enlarged, hyperchromatic nuclei, prominent nucleoli, and abundant cytoplasm (megalocytosis)
Alveolar/Bronchiolar carcinoma	(10%)	In the lung, there was papilliform growth of a pleomorphic population of epithelial cells, which were arranged around a fibrovascular core
B-cell lymphoma	(10%)	There was effacement of the normal splenic architecture by a proliferating population of CD79a-immunopositive lymphoblastic cells
Histiocytic sarcoma	(5%)	Tumour was composed of sheets of large, pleomorphic histiocytic cells with abundant cytoplasm; multinucleated giant cells were common
Metastasis	(20%)	Spleen to liver and lung metastasis, liver (HCC) to lung and liver metastasis

Given that constitutive overexpression of *Cryab* leads to malignancies with late latency ~ 17 months, next we sought to validate this observation by using a 7,12-Dimethylbenz[a]anthracene (hereafter: DMBA)-induced carcinogen model to evaluate differences in the tumorigenic potential of *Cryab*^Wt^, and *Cryab*^Tg^ mice. DMBA is a potent organ-specific carcinogen that serves as a tumor initiator. At postnatal day 5, pups from each genotype were subjected to carcinogen-induced tumorigenesis by topical administration of 1% DMBA [13] and mice were sacrificed at 4 months and analyzed for the development of lung tumors (Additional file 1: Fig. S5a). We found that *Cryab*^Tg^ exhibited a higher number of lung tumor nodules when compared to *Cryab*^Wt^ counterparts suggesting that *Cryab* overexpression can potentiate carcinogen-induced tumorigenesis (Additional file 1: Fig. S5a, b).

### *Cryab* overexpression is correlated with increased angiogenesis and epithelial to mesenchymal transition in tumors

**Fig. 3:**
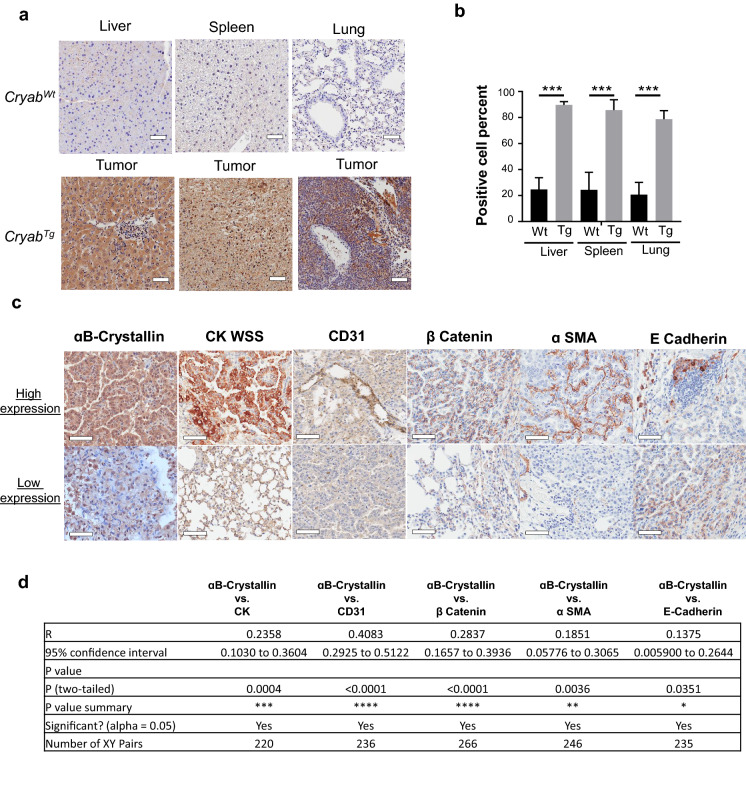
Transgenic expression of *Cryab* correlates with progressive tumorigenesis and expression of tumor markers. **a** Representative microscopic images of normal organs in *Cryab*^wt^ (upper Panel) and *Cryab*^Tg^ (lower Panel) associated malignancy stained with anti-Crystallin antibody (scale bars, 50 µm) **b** Bar chart represents the positive cell count percent of selected tissues for indicated genotypes. **c** Comparison of IHC staining of the high expression of indicated markers in adenocarcinoma (ADC) and low expression in lower grade tumors and normal adjacent tumor tissues of lung from tumor-bearing *Cryab*^Tg^ mice. **d** Correlation comparison between indicated markers shown above

Given the spontaneous formation of tumors and associated metastases in *Cryab*^Tg^ mice; first, we validated the expression level of αB-Crystallin in *Cryab*^Wt^ and *Cryab*^Tg^ tissues. Upon immunohistochemistry staining of αB-Crystallin, we found that it was elevated significantly in liver, spleen, and lung tumors from *Cryab*^Tg^ mice when compared to respective age-matched tissues from *Cryab*^Wt^ mice (Fig. 3a, b). Next, we examined the level of immunohistochemical expression of well documented cancer relevant genes and pathways including TP53, extracellular signal-regulated kinase (ERK) and Protein Kinase B (AKT) in matched tissues of normal and cancer-bearing mice. Expectedly, we observed a higher expression of these markers in tumors from *Cryab*^Tg^ mice compared to age-matched control tissues from *Cryab*^Wt^ (Additional file 1: Fig. S6a). Consistently, we found that cancers in *Cryab*^Tg^ mice had higher levels of p53 when compared to corresponding tissues from *Cryab*^Wt^ mice (Additional file 1: Fig. S6a). The observed increase in p53 expression is most likely an indication of the presence of mutated p53 which might act as a secondary hit to initiate tumorigenesis in *Cryab*^Tg^ mice. Both oncogenic survival signaling pathways, ERK and AKT, might also be activated by *Cryab* in response to stress-induced mechanisms in tumor microenvironment (Additional file 1: Fig. S6a).

Next, we examined whether there is a correlation between the pattern of *Cryab* expression in tumors from *Cryab*^Tg^ mice and the expression of tumor markers in malignant tissues. Tumor types were divided into lymphoma, hepatocellular carcinoma, lung and, liver adenocarcinoma and regions of interest were highlighted based on cytokeratin-wide spectrum screening (CK-WSS) and gross morphology of tumors. The respective histoscore (H-score) for tumor and tumor-adjacent tissues were obtained according to previously published method explained in method Section [14]. Interestingly, there was a strong correlation between cytokeration-wide spectrum screening CK-WSS, a marker of epithelial cancer cells and αB-Crystallin expression in solid tumors (Fig. 3c, d and Additional file 2: Table S1). Furthermore, we found a positive correlation between αB-Crystallin and CD31, a marker of angiogenesis in tumors. There was also a positive correlation between αB-Crystallin and membranous β Catenin which is associated with various carcinomas and epithelial-to-mesenchymal transition (EMT). Moreover, the attenuation of E-cadherin compared to stronger expression of α-smooth muscle actin (SMA) is also linked with EMT. We could not find a significant immunohistochemical correlation between αB-Crystallin expression and Ki67 proliferation marker nor matrix metalloproteinase‑9 (MMP-9) expression in lung adenocarcinomas, however Ki67 in liver lymphoma and MMP9 in hepatocellular carcinoma were correlated with high αB-Crystallin (Additional file 1: Fig. S6b). We also extracted proteins from hepatocellular carcinomas, and adjacent normal tissue from Cryab^Tg^ mice and normal liver tissue from control mice and performed targeted mass spectrometry to quantify EMT (Vimentin, α-Smooth muscle actin (α-SMA) and E-Cadherin) and angiogenesis (VEGFR, CD31, PDGFR) markers to validate whether the elevated level of *Cryab* expression is associated with high expression of these markers. Interestingly, we could find a trend towards increased expression of EMT markers (α-SMA and vimentin) as well as PDGFR as an angiogenesis marker in hepatocellular carcinoma from *Cryab*^Tg^ mice (Additional file 1: Fig. S6c). PDGFs are potent mitogens that transduce signals through PDGFRs and play a key role in the angiogenesis process by up-regulating vascular endothelial growth factors (VEGFs) and modulating the proliferation and recruitment of perivascular cells [15]. The peptides from other angiogenesis markers (VEGF, VEGFR and CD31) were below the detection limit. Taken together, we found a causal link between αB-Crystallin expression and crucial events of tumor initiation and spread in particular angiogenesis and EMT, in agreement with bioinformatic analysis of human tumors shown in (Additional file 1: Fig. S3a and S3C).

Next, we have stained our sections for macrophages (F4/80) as well as Cytotoxic (CD8), and helper (CD4) T-cells. Interestingly, we found a positive correlation between αB-Crystallin and tumor associated macrophages in liver hepatocellular carcinoma sections (Additional file 1: Fig. S6d, left); however, we could not find significant lymphocyte infiltration in these tumors (data not shown). Moreover, in the lung adenocarcinoma, we could find tissue-resident macrophages (most probably Alveolar macrophages, oval shaped) regardless of the level of αB-Crystallin expression (Additional file 1: Fig. S6d, right). Phenotyping of macrophage populations and more in-depth analysis will be a subject of future studies; however, these data are in line with Cibersort analysis of human tumors which revealed that macrophages have a positive correlation with liver hepatocellular carcinoma (LIHC) but not lung adenocarcinoma (LUAD) (Additional file 1: Fig. S3d). Moreover, we did not observe much infiltration of CD8 and CD4 T-cells in LUAD and LIHC which is also consistent with Cibersort analysis (Additional file 1: Fig. S3d). Overall, in hepatocellular carcinoma, macrophages showed a positive correlation with αB-Crystallin expression in our mouse model.

### *Cryab* overexpression promotes colony formation, migration and survival signaling in vitro

**Fig. 4:**
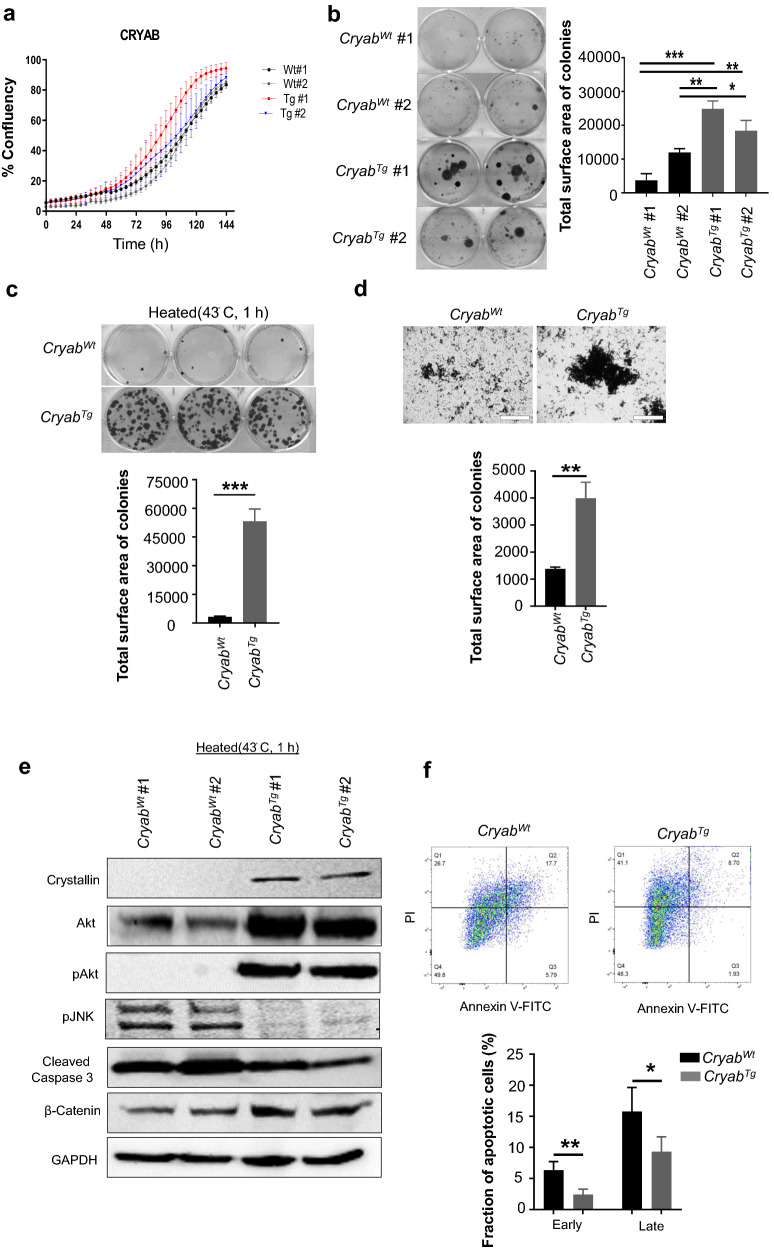
*Cryab* overexpression increases clonogenic survival and transwell migration in vitro. **a** Proliferation of E1A/Ras-transformed MEFs *Cryab*^Wt^ (Wt) and *Cryab*^Tg^ (Tg) measured using IncuCyte (Mean ± SEM, an average of 2 biological repeats in duplicate and 2 independent experiments Student’s t-test, ****P < 0.0001), **b** Representative image (left) and quantification (right) of indicated genotypes of E1A-Ras-transformed MEFs for clonogenic survival (colony formation) assay. Each cell line was seeded at densities of 500 in each well incubated for 14 days, the cells were then processed using the crystal violet stain to visualize the colonies. Quantification performed by analysis of all images **c** Representative image (upper) and quantification (lower) of indicated genotypes of E1ARas-transformed MEFs for clonogenic survival (colony formation) assay as aforementioned above after initial 1 h of heat stress (43ºC). **d** Representative image (upper) and quantification (lower) of wells for indicated genotypes of E1ARas-transformed MEFs for transwell migration assay after an initial 1 h of heat stress (43ºC), (scale bars, 400 µm) Data in (b-d) presented as Mean ± SEM, an average of 3 biological repeats and 3 independent experiments Student’s t-test, ****P < 0.0001). **e** Immunoblot analysis was performed on E1A/Ras-transformed MEFs after exposure to heat stress at 43 °C for 1 h. **f** Quantification of apoptosis by Annexin V/PI flow cytometry in MEF cells of Cryab^Wt^ (Wt) and Cryab^Tg^ (Tg) MEFs after exposure to heat stress at 43 °C for 1 h. Upper panel is representative contour diagram of FACS profile via Annexin V-FITC/PI staining. Live cells which exclude PI and lack Annexin V-FITC binding are confined to left lower quadrant (Q4) of each panel. Dead cells (apoptosis) appear first in the right lower quadrant(Q3) detected with Annexin V-FITC and then in the upper right quadrant (Q2; late apoptotic cells) as a result of the loss of cell membrane integrity and are positive for both Annexin V-FITC binding and for PI uptake. Lower panel, Bar graph demonstrates the percent of early and late apoptotic cells in Cryab^Wt^ (Wt) and Cryab^Tg^ (Tg) MEFs after exposure to heat stress at 43 °C for 1 h. Data presented as Mean ± SD, an average of 3 biological repeats and 2 independent experiments Student’s t-test, **P < 0.01)

The identification that *Cryab* overexpression causes spontaneous tumor formation in multiple organs and is linked to essential oncogenic markers in vivo prompted us to evaluate the molecular mechanism underpinning these phenotypes. Consequently, MEFs from *Cryab*^Wt^, and *Cryab*^Tg^ transgenic mice (Additional file 1: Fig. S7a) were generated and transformed using a construct expressing E1A/RasV12 oncogenes [16] to evaluate signaling roles of αB-Crystallin in in vitro transformed-fibroblasts. Heat-shock stress (43 ℃) was used to monitor the induction of *Cryab* expression at various time-points (Additional file 1: Fig. S7b). As *Cryab*^Tg^ MEFs demonstrated significantly higher expression of αB-Crystallin compared to *Cryab*^Wt^ MEFs within 1 h after heat-shock stress, this time-point was utilized for subsequent experiments. Next, we compared the proliferation potential of MEFs using IncuCyte^®^ live cell analysis for 6 days. Despite marginally increased growth in one of the *Cryab*^Tg^ MEF lines, no significant differences between the proliferation rate of *Cryab*^Wt^ and *Cryab*^Tg^ MEFs were observed (Fig. 4a). However, *Cryab*^Tg^ MEFs had enhanced clonogenic potential in vitro as assessed by colony-forming ability compared to *Cryab*^Wt^ counterparts (Fig. 4b) and the differences in clonogenic survival were accentuated after heat-shock treatment (Fig. 4c). Moreover, the transwell migration assay demonstrated that *Cryab*^Tg^ MEFs showed increased migratory potential compared to *Cryab*^Wt^ MEFs following heat-shock treatment (Fig. 4d).

Next, we investigated possible signaling pathways altered by the overexpressed αB-Crystallin in MEFs. Importantly, β Catenin had elevated expression in *Cryab*^Tg^ MEFs compared to control MEFs which is consistent with IHC staining in tumors which indicated the activation of the Wnt signaling pathway. Additionally, we found that total AKT and phosphorylated AKT (Ser 473) were both upregulated (Fig. 4e). The PI3K/AKT is a major survival pathway that plays multiple roles in cellular processes such as regulation of cell proliferation, apoptosis, metabolism, and cell migration. To further examine the role of αB-Crystallin in apoptosis evasion, we examined the induction of apoptotic pathways following heat shock. We found that the expression of the pro-apoptotic phosphorylated JNK (pJNK T184/Y185) and apoptotic marker cleaved caspase-3 were both downregulated in the *Cryab*^Tg^ lines (Fig. 4e). Furthermore, annexin V staining to identify and quantify the early and late apoptotic cells revealed that *Cryab*^Tg^ MEF underwent less apoptosis in response to heat shock stress compared to Cryab^Wt^ counterparts (Fig. 4f). Overall, *Cryab* overexpression promotes clonogenic potential, increases cell migration and cell survival signaling and inhibits stress-induced apoptosis.

### *Cryab* overexpression upregulates metastatic and oncogenic markers

**Fig. 5:**
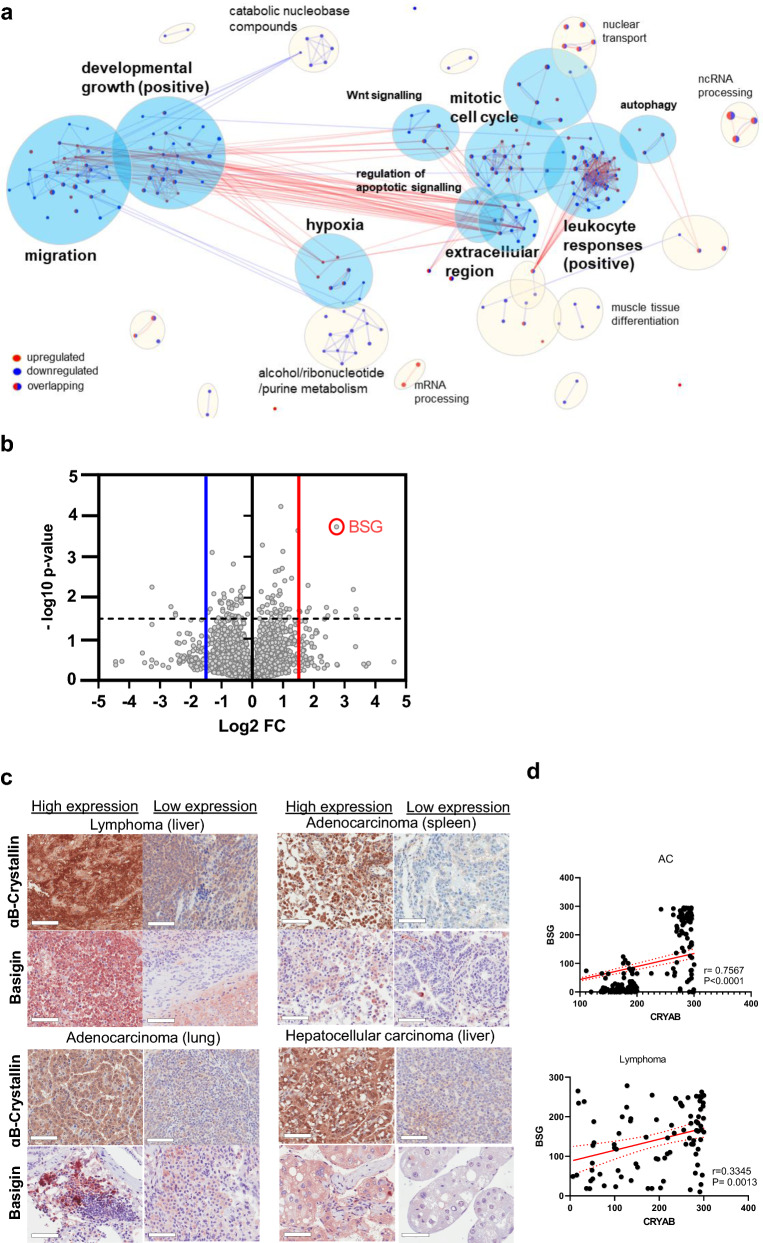
Mass spectrometry (MS)-based proteomics analysis reveales Basigin upregulation in *Cryab* overexpressed cells. **a** Biological pathway enrichment analysis of the proteomic data comparing *Cryab*^Tg^ E1A/Ras-transformed MEFs to *Cryab*^WT^ MEFs revealed differentially regulated pathways visualized using Cytoscape. Upregulated (red) and downregulated (blue) node proteins in clusters are indicated. **b** Volcano plot showing up/downregulated proteins (*Cryab*^Tg^/*Cryab*^WT^) in the proteomic data with corresponding fold changes and p-values. Highlighted BSG had the highest p-value with ~ sevenfold upregulation. **c** Representative IHC images for comparison of Cryab (high and low expressing areas) and BSG expression in Lymphoma (Lym) liver, adenocarcinoma (ADC) of lung, liver and hepatocellular carcinoma (HCC) of tumour-bearing *Cryab*^Tg^ mice, (scale bars, 50 µm). **d** Correlation (linear regression) between expression of *Cryab* and *Bsg* in adenocarcinoma and lymphoma of tumor-bearing *Cryab*^Tg^ mice

To investigate the signatures enhancing the oncogenic potential of *Cryab*^Tg^ MEFs, we evaluated the proteome landscape of *Cryab*^Wt^ and *Cryab*^Tg^ MEFs. The mass spectrometry‐based proteomic analysis revealed 135 downregulated and 104 upregulated proteins > log_2_ (± 0.6) in *Cryab*^Tg^ MEFs over *Cryab*^Wt^ after 1 h heat-shock (43 ℃) stimulation. Gene enrichment analysis identified differential regulation in biological pathways such as epithelial morphogenesis/migration (migration), developmental growth, hypoxia, mitotic cell cycle, response to leukocytes (activation and proliferation in immune system process), regulation of apoptosis, extracellular remodeling and wnt signaling in *Cryab*^Tg^ MEFs (Fig. 5a). The volcano plot of downregulated and upregulated proteins revealed the plasma membrane immunoglobulin-like protein Basigin (BSG, CD147), gained the highest fold change and p value among the others (Fig. 5b). Basigin is overexpressed in many different human cancer types and plays a crucial role in regulation of cancer cell invasion, migration and angiogenesis [17]. Basigin is strongly connected to protein clusters for epithelial morphogenesis/migration, developmental growth and immunity. Basigin with linked upregulated proteins enhancing metabolism and growth such as growth factor HBEGF, glucose transporter SLC2A1 and proto-oncogene JUNB, combined with downregulated protective pathways containing donor against oxidative stress G6PDX, redox chaperone PARK7 (purine metabolism) and DNA repair enzyme APEX1 (catabolic nucleobase compounds) [17], likely provide an advantage for overall oncogenic growth and transformation (Fig. 5a, Additional file 1: Fig. S8a). Supporting the biological pathway enrichment analysis, the gene annotation of upregulated genes linked most of them to metastasis and tumorigenic phenotypes (Additional file 3: Table S2). Intriguingly, top five upregulated genes are all linked to regulation of oncogenesis and metastasis (Additional file 1: Fig. S8b) and pan cancer analysis of TCGA dataset revealed that these genes have co-occurrence of expression in cancers including CRYAB with BSG and HELLS (Additional file 1: Fig. S8c). Further supporting relevance to human cancer development, CRYAB expression levels are significantly higher in metastatic contexts in a range cancer types (Additional file 1: Fig. S9a–i). Additionally, CRYAB expression significantly correlates with EMT in various cancer types and with angiogenesis in 14 of 22 (64%) cancer types of epithelial origin as already shown in Additional file 1: Fig. S3.

**Fig. 6:**
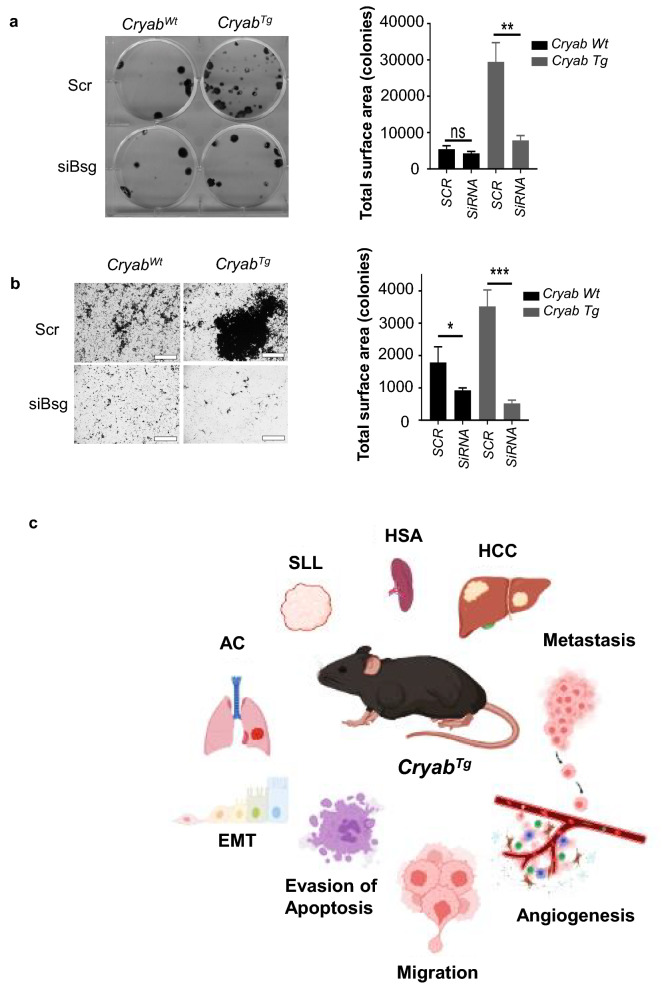
Basigin (BSG) inhibition reduces colony formation and cell migration. **a** colony formation assay (**b**) transwell migration assay (lower) in E1A/Ras-transformed MEFs, generated from *Cryab*^wt^ and *Cryab*^Tg^ mice, treated with Scr (scrambled) and siRNA (# 6) against BSG. Scale bars, 400 µm, data presented as Mean ± SEM, an average of 3 biological repeats and 3 independent experiments. Student’s t-test was employed for statistical significance, ****P < 0.0001). **c** The graphical abstract of *Cryab*^Tg^ mouse model shows spontaneous tumorigenesis and metastasis in vivo leading to different cancer types such as Hepatocellular carcinoma (HCC), Hemangiosarcoma (HSA), Small lymphocytic lymphoma (SLL), and Adenocarcinoma (AC) due to regulation of several pro-tumorigenic mechanisms including EMT, evasion of apoptosis, angiogenesis and migration (illustrated by BioRender)

To validate these finding, we performed immunostaining of Basigin in tumor tissues of *Cryab*^Tg^ mouse model. These data validated that Basigin protein expression positively correlates with *Cryab* expression in tumors and adjacent tumor tissues (Fig. 5c, d). Next, we performed siRNA mediated knockdown of *Bsg* in MEFs and the gene knockdown efficiency was examined by immunoblotting (Additional file 1: Fig. S9j). Interestingly, Bsg-depletion (using siRNA #6) led to the reduction of colony formation in *Cryab*^Tg^ MEFs (Fig. 6a). Furthermore, Bsg-depletion significantly decreased migration in *Cryab*^Wt^ and *Cryab*^Tg^ MEFs by transwell migration assay (Fig. 6b). Collectively, the data suggests that some of the phenotypic effects of *Cryab* overexpression might-in part be mediated via upregulation of Bsg expression in our murine and cellular models.

In conclusion, we have characterized the mouse model of *Cryab*^Tg^ which led to the spontaneous formation of tumors and associated metastasis due to pro-tumorigenic alteration in promoting survival signaling and migration-EMT and evasion of apoptosis (Fig. 6c).

## Discussion

We find that transcriptional upregulation of αB-Crystallin is widespread in human cancers and correlates with poor patient prognosis. To understand the contribution of αB-Crystallin to de novo tumorigenesis, we generated and characterized a mouse model that constitutively overexpresses *Cryab* in multiple tissues. *Cryab* overexpressing mice (homozygous *Cryab*^*Tg*^) develop a wide spectrum of solid and hematological tumors with an approximate 50% incidence rate and metastatic potential, although with late latency. Notably, in the time-frame of the study only one control mice died due to idiopathic cause. Long latencies are characteristics of many other mouse models which suggest that cells may need to acquire time-dependent genetic and/or epigenetic changes before malignant transformation occurs [18, 19]. Strikingly, we found higher p53 protein levels, which are most likely an indication of *Trp53* mutation in representative *Cryab*^Tg^ tumor tissues, suggesting that Tp53 might be a critical secondary hit required for tumor initiation observed in the homozygous *Cryab*^Tg^ mice. The tumors were further characterized histopathologically which identified lung adenocarcinoma, lung metastasis, hepatocellular carcinoma, and lymphoma as the major types of malignancies. Furthermore, *Cryab*^Tg^ mice also showed the increased carcinogen-DMBA induced tumor load. Like *Cryab* (also known as *HSPB5*), other heat shock proteins have been functionally linked to spontaneous tumorigenesis, for example, transgenic mice expressing human HSP70 developed lung and lymph node tumours before 18 months of age [20]. In addition, HSP27 increased the tumorigenicity of rat colon adenocarcinoma in a syngeneic model [21]. Furthermore, HSP90 is similarly up-regulated in a wide variety of cancers and inhibitors of HSP90 are currently in clinical trials as chemotherapeutic drugs [22].

In our transgenic mouse model, we found elevated levels of *Cryab* expression in *Cryab*^Tg^ tumors compared to adjacent non-tumor tissues and age-matched normal tissues from *Cryab*^Wt^ [2]. *Cryab* is a multi-functional protein with postulated roles in the regulation of cell architecture, apoptosis, and autophagy through interactions with a multitude of its substrate proteins [23]. Therefore, there are several potential mechanisms by which *Cryab* may promote tumorigenesis. We have found that this may be caused by elevated levels of AKT and ERK survival pathways in *Cryab*^Tg^ tissues (both tumors and adjacent tissues) compared to tissues from *Cryab*^Wt^ mice. Interestingly, focusing on solid tumors from mice, *Cryab* overexpression was correlated with cytokeratin-screening (CK-WSS), which reflects tumor cell activity and is considered a tumor marker. Furthermore, we found a correlation between overexpressed *Cryab* and both angiogenesis and EMT markers in *Cryab*^Tg^ tumors, which may both enhance tumor phenotypes. The negative consequence of *Cryab* depletion on tumor angiogenesis has been established in in vitro studies using cancer cell lines although, increased *Cryab* expression has not previously been functionally linked to increased angiogenesis in tumors [24, 25]. In terms of tumor infiltration, we could only find a correlation of tumor associated macrophage infiltration in areas of high expression of *Cryab*^Tg^ in hepatocellular carcinoma tumors which again is in line with Cibersort analysis of TCGA data for human liver hepatocellular carcinoma. These results are consistent with our pan-cancer analysis of TCGA data which revealed strong correlation of high *CRYAB* expression with angiogenesis, EMT and metastasis in a variety of human cancer types, suggesting that our observations are relevant to cancer development in patients.

To complement and extend prior knowledge of αB-Crystallin biology, we performed further phenotypic analysis of E1A/RAS-transformed MEFs derived from *Cryab*^Tg^ and *Cryab*^Wt^ mice. Notably, we show that overexpression of *Cryab* in transformed MEFs is sufficient to impart tumorigenic properties in vitro including increased clonogenic capacity, and migratory and invasive potential. Functional consequences of *Cryab* overexpression were examined by proteomics analysis of the transformed MEFs of both genotypes, which also pointed to pleiotropic roles of *Cryab* in the regulation of many metastatic and oncogenic proteins. The most significantly upregulated protein was Basigin, which we found had a positive correlation with the expression of *Cryab*, validated both in tumors compared to adjacent normal tissues and in vitro in MEFs. Basigin is also a transmembrane protein that mainly functions in metabolic pathways, such as glycolysis, but its overexpression is associated with several pathologies including cancer [26–29]. In particular, its overexpression is correlated with worse overall survival in acute myeloid leukaemia, and non-small-cell lung cancer (NSCLC) and plays important role in regulation of cancer cell proliferation, invasion and metastasis [27, 29, 30]. Its cancer connection is mostly linked to its capacity to regulate expression/activity of monocarboxylate transporters, matrix metalloproteinases and PI3K and MAPK pathways. It also functions as a key mediator of inflammatory/immune response. Interestingly, the knockdown of Basigin reduced the oncogenic colony formation ability and migratory potential of *Cryab*^Tg^ MEFs, suggesting that some of the phenotypic effects of *Cryab* overexpression might-in part be mediated by regulation of Basigin expression.

We also found elevated levels of AKT and pAKT in *Cryab*^Tg^ MEFs, consistent with our observations in *Cryab*^Tg^ tumors, and this might be a potential cause of spontaneous tumorigenesis in our model. The PI3K/AKT pathway is involved in various cellular processes, including the promotion of cell survival, and cell cycle and is often altered in various cancers [31]. AKT can inhibit apoptosis by phosphorylating and inhibiting pro-apoptotic proteins such as Bad, Bim and caspase 9 [32]. It can also promote the cell cycle progression by inhibiting both Cyclin-Dependent Kinase (CDK) degradation and CDK Inhibitors expression, allowing cell cycle activation [31]. The upregulation of pAKT and total AKT by *Cryab* overexpression are consistent with another in vitro model in particular near-normal mammary epithelial cell line, MCF10A, in which *Cryab*-overexpression promoted malignant transformation and growth of mouse mammary xenograft tumors through regulation of AKT [6]. On the other hand, JNK was found to be downregulated in *Cryab*^Tg^ MEFs. JNK directly induces apoptosis by activating pro-apoptotic proteins such as the previously mentioned Bad and Bim and inhibiting anti-apoptotic proteins such as Bcl-xL. JNK has been reported to antagonize PI3K/AKT pathway, and the reduced apoptosis observed in our model may partly potentiate the tumor formation where JNK antagonism of Akt-mediated survival signals is suppressed. Evasion of apoptosis is a well-described hallmark of cancer resulting in cellular resistance to conventional chemotherapeutic agents [1]. Indeed, resistance to apoptosis during myocardial ischemia was observed in mice overexpressing *Cryab* in cardiomyocytes [33]. Altogether, these results show that the overexpression of *CRYAB* leads to the promotion of cell survival after induction of cellular stress, which is consistent with its oncogenic potential. This may occur via the activation of the AKT pathway and inhibition of the JNK pathway. The promotion of cell survival was validated by the decreased levels of cleaved caspase 3.

## Conclusion

In summary, this is the first report to our knowledge that has causally linked *Cryab* overexpression to oncogenesis. Our data demonstrate that *Cryab* overexpression beyond a critical level is self-sufficient to induce a wide spectrum of spontaneous tumors in aged mice. We have found that this may be caused by an upregulation of the oncogenic AKT survival pathways, and modulation of the tumor suppressor JNK pathway. Notably, overexpression of *Cryab* in transformed MEFs is sufficient to impart tumorigenic properties in vitro and further studies are required to enhance the knowledge of how *Cryab* regulates these phenotypes, especially in a hypoxic and oxidative stress environment of tumor cells. Since *CRYAB* overexpression is a common phenomenon in human cancers, we have initially generated a model to study its involvement in multiple cancer types. In future, conditional tissue specific overexpression of *Cryab* and crossing of *Cryab*-conditional mouse line with other cancer-prone models such as KrasG12D expression, PTEN and/or p53 loss will allow detailed mechanistic evaluation of its function in a given cancer type.

## Supplementary Information

**Additional file 1. Figure S1.** *CRYAB* is frequently gained copy number in many cancer types and these copy number changes are correlated with Crystalin expression in some cancers. (a) Chart shows the percentages of copy number amplification (red) or copy number gain (pink) of *CRYAB* across various cancers using the cBioPortal for Cancer Genomics. (b) Correlation between *CRYAB* copy number and expression level across cancer types. Comparison between *CRYAB* copy number (x-axis) and *CRYAB* expression level (y-axis) in tumour samples from The Cancer Genome Atlas (TCGA) across 14 cancer types, as indicated. X-axis labels respectively refer to: *CRYAB* copy number deep loss (-2), loss (-1), copy number neutral (0), gain (1) and amplification (2). P values and p value abbreviations: Pearson correlation. **Figure S2.**CRYAB higher expression is associated with poor survival in multiple cancer types. Kaplan-Meier survival analysis for TCGA cancer type indicated below the chart for the overall survival. Patients were split into two groups of high (red) and low (black) CRYAB expression levels. Numbers of patients at risk are shown at the bottom. **Figure S3.** *CRYAB* overexpression correlates with deregulation of signaling, oncogenic features and tumor infiltration across some cancers. (a–d) The heatmaps show the correlations between *CRYAB* gene expression levels and variables (determined via the integration of multi-platform data from several types of ‘omics’ analyses) listed on the y-axis in up to 32 cancer types, whose abbreviations are listed on the top x-axis based on the methodology previously described 9 ; for all analysis (a-d): each tile in the heatmaps shows the Spearman correlation between the gene expression levels (mRNA levels from The Cancer Genome Atlas (TCGA) RNAseq datasets) and the variables listed below. The color of each tile reflects the Spearman correlation coefficient (r), as indicated by the color key on the right. Each tile shows the Spearman p value abbreviations in the following format: blank tiles: p > 0.05; *, p < 0.05; **, p < 0.01; ***, p < 0.001; ****, p < 0.0001. Cancer type abbreviations: ACC, Adrenocortical Cancer; BLCA, Bladder Cancer; BRCA, Breast Cancer; CESC, Cervical Cancer; CHOL, Cholangiocarcinoma (bile duct cancer); COAD, Colon Cancer; DLBC, Large B-cell Lymphoma; ESCA, Esophageal Cancer; GBM, Glioblastoma; HNSC, Head and Neck Cancer; KICH, Kidney Chromophobe; KIRC, Kidney Clear Cell Carcinoma; KIRP, Kidney Papillary Cell Carcinoma; LGG, Lower Grade Glioma; LIHC, Liver Cancer; LUAD, Lung Adenocarcinoma; LUSC, Lung Squamous Cell Carcinoma; MESO, Mesothelioma; OV, Ovarian Cancer; PAAD, Pancreatic Cancer; PCPG, Pheochromocytoma & Paraganglioma; PRAD, Prostate Cancer; READ, Rectal Cancer; SARC, Sarcoma; SKCM, Melanoma; STAD, Stomach Cancer; TGCT, Testicular Cancer; THCA, Thyroid Cancer; THYM, Thymoma; UCEC, Endometrioid Cancer; UCS, Uterine Carcinosarcoma; UVM, Ocular melanoma. **Figure S4.** Protein alignment and mouse model target validation. (a) Alignment of human (NP_001876, upper in red) and mouse (NP_034094.1, lower in blue) ɑB-Crystallin protein sequences. The spaces indicate sequence mismatch and a + indicates a positive match on the scoring matrix and ɑB-crystallin domain is underlined in the consensus. (b) PCR genotyping showing *Cryab*^Wt/Tg^ (Het), *Cryab*^Wt/Wt^ (Wt) and *Cryab*^Tg/Tg^ (Tg) genotypes. (c) Immunoblot analysis of Crystallin protein expression of *Cryab*^Wt/Wt^, *Cryab*^Wt/Tg^, and *Cryab*^Tg/Tg^ mouse tissues as indicated in the figure. β-actin was used as a loading control. (d) Immunoblot analysis of Crystallin protein expression of tumor and adjacent tumor tissues from two lungs of *Cryab*^Tg/Tg^ mice. Vinculin was used as a loading control. **Figure S5.** *CRYAB* overexpression increases carcinogen (DMBA)-induced tumorigenesis (a) Chart shows number of nodules from dissected lungs of DMBA-treated *Cryab*^Wt/Wt^, *Cryab*^Wt/Tg^, and *Cryab*^Tg/Tg^ mice, the outliers were removed using the ROUT method (Q = 1%) based on the False Discovery Rate (FDR). (b) representative image of Bouin's solution fixed lungs from each genotype, the visible lung tumors (nodules) were counted by checking the surface of each lung lobe (upper) and H&E stained tissue from *Cryab*^Wt/Wt^ and *Cryab*^Tg/Tg^ was used to validate the tumor counting ( lower), arrows show the nodules or tumors, the bigger size of tumors is evident in CryabTg mice.. **Figure S6.** IHC on tumors and normal tissues and targeted mass spectrometry (MS). (a) IHC staining compares the expression level of indicated markers in malignant tissue of *Cryab*^Tg/Tg^ with age and tissue matched control from *Cryab*^Wt/Wt^, (b) IHC staining compares expression of indicated markers in Crystallin high and low expression regions of malignant tissue from *Cryab*^Tg^ mice. (c) Chart shows the peak area of indicated peptides (obtained based on targeted-MS) ɑ-SMA (VAPEEHPTLLTEAPLNPK), Vimentin (FADLSEAANR ) and PDGFR (YGDLVDYLHR) in each group of *Cryab*^Wt^ liver (black), normal liver tissue from *Cryab*^Tg^ (blue), liver tumor from *Cryab*^Tg^ (red). All protein lysates obtained from FFPE tissue sections annotated by pathologist for tumor and normal tissues. (d) IHC staining compares the expression level of indicated markers: Crystallin (green), F4/80 (macrophages, yellow) DAPI (nucleus, blue) in Crystallin high and low expression regions of liver hepatocellular carcinoma section (left) and lung adenocarcinoma sections of *Cryab*^Tg/Tg^ mice(right), Boxed areas are shown at higher magnification where indicated (Scale bars, 100 µm). **Figure S7.** Genotyping and WB on MEFs. (a) PCR genotyping showing *Cryab*^Wt/Wt^ (Wt) and *Cryab*^Tg/Tg^ (Tg) E1A/Ras transformed MEFs genotypes (b) Immunoblot analysis of Crystallin protein expression of *Cryab*^Wt/Wt^, and *Cryab*^Tg/Tg^ E1A/Ras transformed MEFs after heat-shock (43̊ C for 1 hour) following by time course incubation as indicated in the figure. GAPDH was used as a loading control. **Figure S8.** Biological process annotation. (a) Functional annotation and pathway enrichment analysis of differentially regulated proteins of the proteomic data comparing Cryab Tg E1ARas-transformed MEFs to *Cryab*^WT^ MEFs for either upregulated ( green) or downregulated (yellow), Data were normalized to the enrichment ratio. Data was plotted using default parameters of DAVID (the database for annotation, visualization and integrated discovery) (b) The table shows the top five highest upregulated genes. (c) co-occurance analysis of *CRYAB* gene and the top five highest upregulated genes from TCGA pan-cancer. **Figure S9.** CRYAB expression is associated with metastasis in human tumors. (a) CRYAB expression levels in normal cervix and early-stage cervical tumors. (b) CRYAB expression levels in primary and metastatic renal cell carcinoma. (c-f) CRYAB expression levels in primary tumors without and with metastasis in, respectively: (c) hepatocellular carcinoma, (d) pancreatic cancer, (e) breast cancer and (f) colorectal cancer. (g) CRYAB expression levels in pancreatic cancer locally metastasized to pancreas or distantly metastasized to muscle. (h) CRYAB expression levels in prostate cancer locally metastasized to lymph node or distantly metastasized to liver. (i) CRYAB expression levels in lung cancer locally metastasized to lymph node or distantly metastasized to brain. Error bars indicate means and standard errors. P values: two-sided t tests. (j) Immunoblot analysis of siRNA mediated knockdown of Basigin was performed on E1A/Ras-transformed MEF using Scrambled as a control and two siRNA sequences targeting exon 2 and exon 6 of Basigin, vinculin served as a loading control.

**Additional file 2. Table S1.** Correlation analysis.

**Additional file 3. Table S2.** List of the upregulated genes and annotation.

**Additional file 4.** Supplementary methodology:TCGA data analysis and targeted mass-spectrometry. 

## Data Availability

The mass spectrometry proteomics data have been deposited to the Mendeley Data, Elsevier with the dataset identifier DOI:10.17632/kp6dk64gn5.1.

## Abbreviations

BSG: Basigin/CD147
CK-WSS: Cytokeratin-wide spectrum screening
CRYAB: Alpha Crystallin B-chain/ αB-Crystallin
DMBA: 7,12-Dimethylbenz[a]anthracene
EMT: Epithelial-to-mesenchymal
ER: Endoplasmic reticulum
MEFs: Mouse embryonic fibroblasts
MMP-9: Matrix metalloproteinase‑9
Tg: Transgenic
UPR: Unfolded protein response
Wt: Wild-type
Α-SMA: α-Smooth muscle actin
