# Supplementary material for: Alpha-B-Crystallin overexpression is sufficient to promote tumorigenesis and metastasis in mice
Source: Exp Hematol Oncol. 2023 Jan 9;12:4. doi: 10.1186/s40164-022-00365-z (PMC9830749; doi:10.1186/s40164-022-00365-z)
Supplement: Supplementary file 1 — Additional file 1. Figure S1. CRYAB is frequently gained copy number in many cancer types and these copy number changes are correlated with Crystalin expression in some cancers. (a) Chart shows the percentages of copy number amplification (red) or copy number gain (pink) of CRYAB across various cancers using the cBioPortal for Cancer Genomics. (b) Correlation between CRYAB copy number and expression level across cancer types. Comparison between CRYAB copy number (x-axis) and CRYAB expression level (y-axis) in tumour samples from The Cancer Genome Atlas (TCGA) across 14 cancer types, as indicated. X-axis labels respectively refer to: CRYAB copy number deep loss (-2), loss (-1), copy number neutral (0), gain (1) and amplification (2). P values and p value abbreviations: Pearson correlation. Figure S2.CRYAB higher expression is associated with poor survival in multiple cancer types. Kaplan-Meier survival analysis for TCGA cancer type indicated below the chart for the overall survival. Patients were split into two groups of high (red) and low (black) CRYAB expression levels. Numbers of patients at risk are shown at the bottom. Figure S3. CRYAB overexpression correlates with deregulation of signaling, oncogenic features and tumor infiltration across some cancers. (a–d) The heatmaps show the correlations between CRYAB gene expression levels and variables (determined via the integration of multi-platform data from several types of ‘omics’ analyses) listed on the y-axis in up to 32 cancer types, whose abbreviations are listed on the top x-axis based on the methodology previously described 9 ; for all analysis (a-d): each tile in the heatmaps shows the Spearman correlation between the gene expression levels (mRNA levels from The Cancer Genome Atlas (TCGA) RNAseq datasets) and the variables listed below. The color of each tile reflects the Spearman correlation coefficient (r), as indicated by the color key on the right. Each tile shows the Spearman p value abbreviations in th [file 40164_2022_365_MOESM1_ESM.pdf]

Additional file 1

Fig. S1

a

b

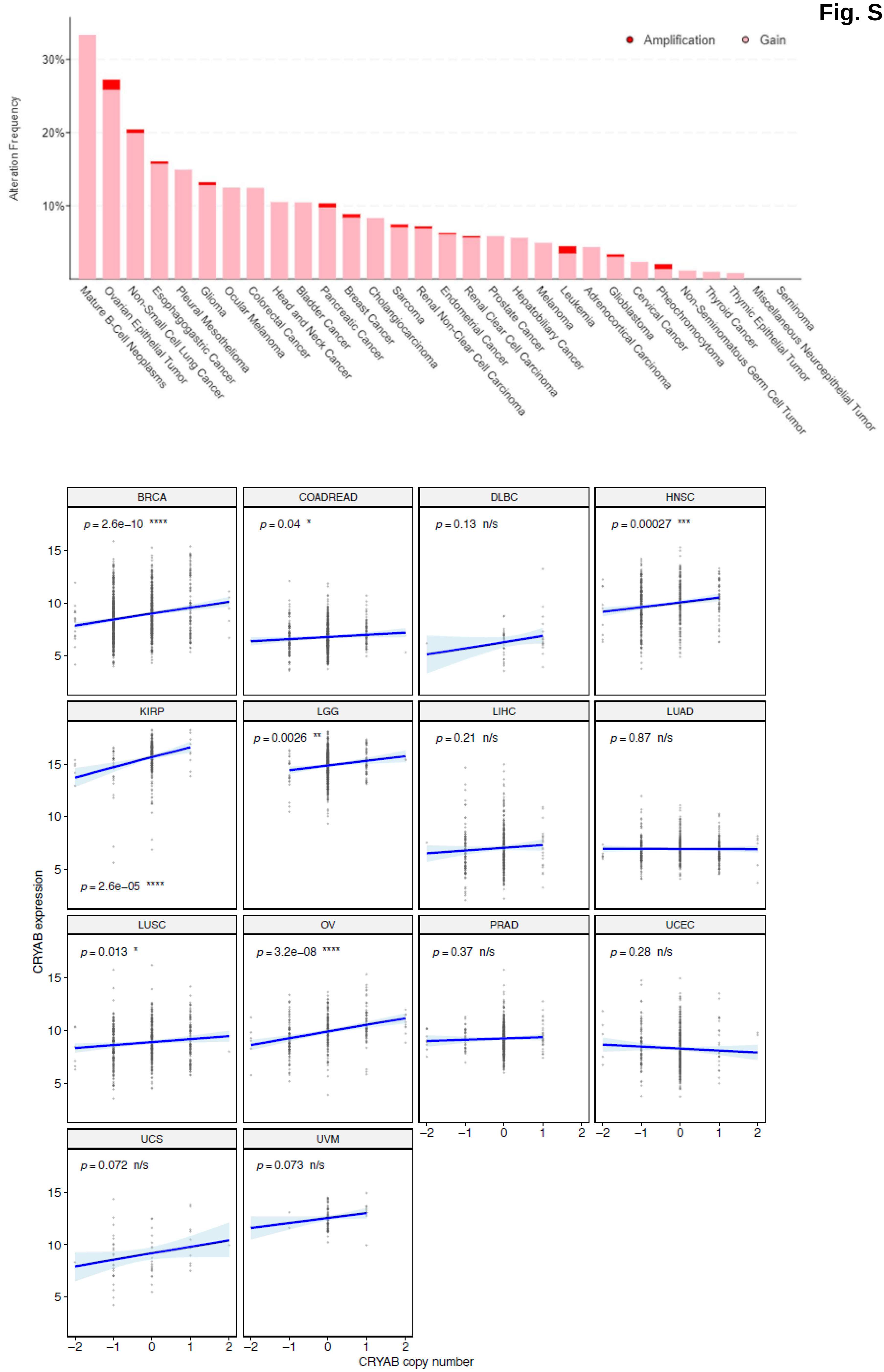

Amplification
Gain
Alteration Frequency
30%
20%
10%
Mature B-Cell Neoplasms
Ovarian Epithelial Tumor
Non-Small Cell Lung Cancer
Esophagogastric Cancer
Pleural Mesothelioma
Glioma
Ocular Melanoma
Colorectal Cancer
Head and Neck Cancer
Bladder Cancer
Pancreatic Cancer
Breast Cancer
Cholangiocarcinoma
Sarcoma
Renal Non-Clear Cell Carcinoma
Endometrial Cancer
Renal Clear Cell Carcinoma
Prostate Cancer
Hepatobiliary Cancer
Melanoma
Leukemia
Adrenocortical Carcinoma
Glioblastoma
Cervical Cancer
Pheochromocytoma
Non-Seminomatous Germ Cell Tumor
Thyroid Cancer
Thymic Epithelial Tumor
Miscellaneous Neuroepithelial Tumor
Seminoma
BRCA
p = 2.6e−10 ****
COADREAD
p = 0.04 *
DLBC
p = 0.13 n/s
HNSC
p = 0.00027 ***
KIRP
p = 2.6e−05 ****
LGG
p = 0.0026 **
LIHC
p = 0.21 n/s
LUAD
p = 0.87 n/s
LUSC
p = 0.013 *
OV
p = 3.2e−08 ****
PRAD
p = 0.37 n/s
UCEC
p = 0.28 n/s
UCS
p = 0.072 n/s
UVM
p = 0.073 n/s
CRYAB expression
CRYAB copy number

Fig. S2

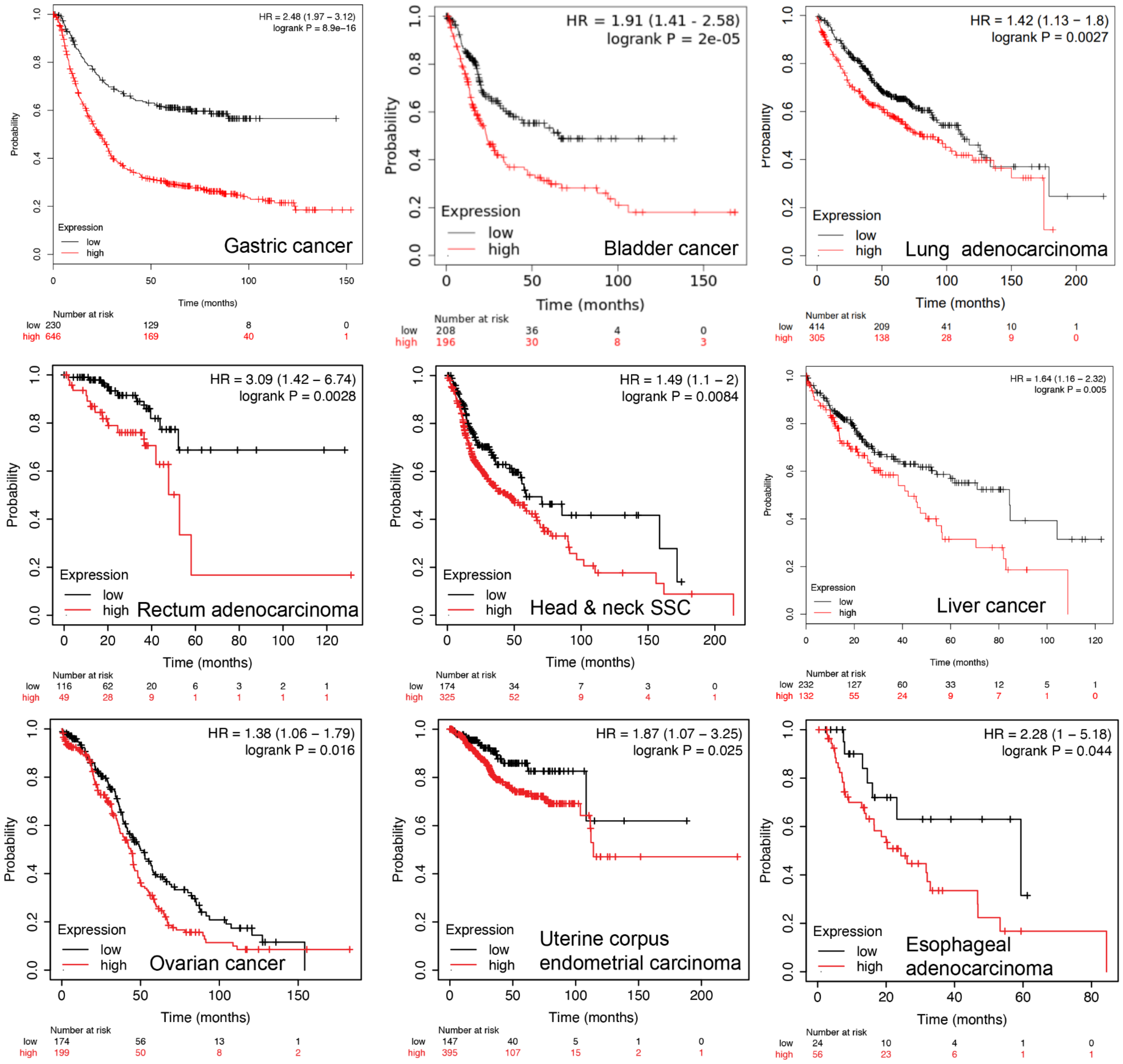
HR = 2.48 (1.97 – 3.12)
logrank P = 8.9e−16
Gastric cancer
Expression
low
high
Probability
Time (months)
Number at risk
low 230 129 8 0
high 646 169 40 1
HR = 1.91 (1.41 - 2.58)
logrank P = 2e-05
Bladder cancer
Expression
low
high
Probability
Time (months)
Number at risk
low 208 36 4 0
high 196 30 8 3
HR = 1.42 (1.13 – 1.8)
logrank P = 0.0027
Lung adenocarcinoma
Expression
low
high
Probability
Time (months)
Number at risk
low 414 209 41 10 1
high 305 138 28 9 0
HR = 3.09 (1.42 – 6.74)
logrank P = 0.0028
Rectum adenocarcinoma
Expression
low
high
Probability
Time (months)
Number at risk
low 116 62 20 6 3 2 1
high 49 28 9 1 1 1 1
HR = 1.49 (1.1 – 2)
logrank P = 0.0084
Head & neck SSC
Expression
low
high
Probability
Time (months)
Number at risk
low 174 34 7 3 0
high 325 52 9 4 1
HR = 1.64 (1.16 – 2.32)
logrank P = 0.005
Liver cancer
Expression
low
high
Probability
Time (months)
Number at risk
low 232 127 60 33 12 5 1
high 132 55 24 9 7 1 0
HR = 1.38 (1.06 – 1.79)
logrank P = 0.016
Ovarian cancer
Expression
low
high
Probability
Time (months)
Number at risk
low 174 56 13 1
high 199 50 8 2
HR = 1.87 (1.07 – 3.25)
logrank P = 0.025
Uterine corpus endometrial carcinoma
Expression
low
high
Probability
Time (months)
Number at risk
low 147 40 5 1 0
high 395 107 15 2 1
HR = 2.28 (1 – 5.18)
logrank P = 0.044
Esophageal adenocarcinoma
Expression
low
high
Probability
Time (months)
Number at risk
low 24 10 4 1 0
high 56 23 6 1 1

Fig. S3

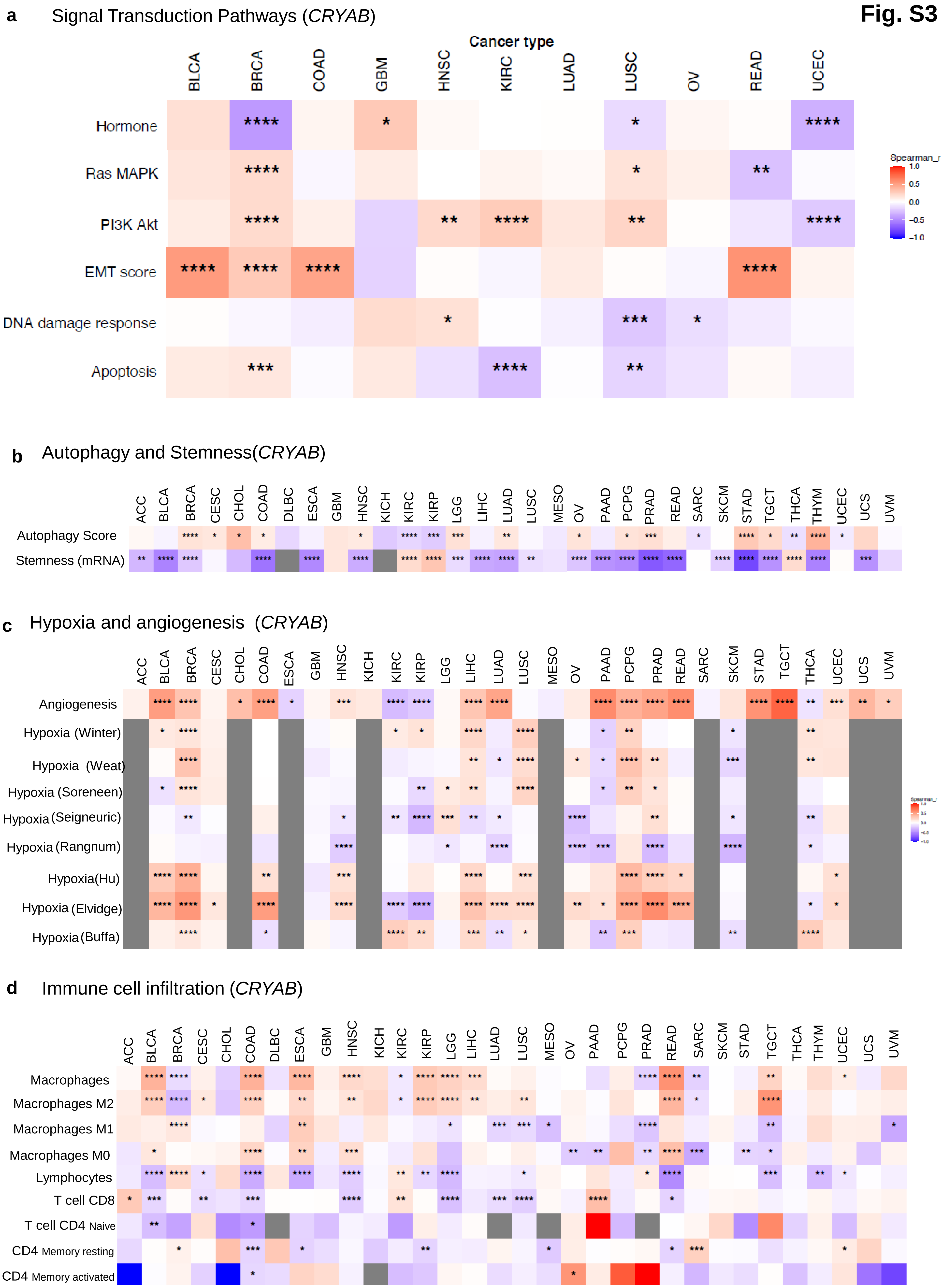
a Signal Transduction Pathways (CRYAB)
Cancer type
BLCA
BRCA
COAD
GBM
HNSC
KIRC
LUAD
LUSC
OV
READ
UCEC
Hormone
Ras MAPK
PI3K Akt
EMT score
DNA damage response
Apoptosis
Spearman_r
1.0
0.5
0.0
−0.5
−1.0
b Autophagy and Stemness(CRYAB)
ACC
BLCA
BRCA
CESC
CHOL
COAD
DLBC
ESCA
GBM
HNSC
KICH
KIRC
KIRP
LGG
LIHC
LUAD
LUSC
MESO
OV
PAAD
PCPG
PRAD
READ
SARC
SKCM
STAD
TGCT
THCA
THYM
UCEC
UCS
UVM
Autophagy Score
Stemness (mRNA)
c Hypoxia and angiogenesis (CRYAB)
Angiogenesis
Hypoxia (Winter)
Hypoxia (Weat)
Hypoxia (Soreneen)
Hypoxia (Seigneuric)
Hypoxia (Rangnum)
Hypoxia(Hu)
Hypoxia (Elvidge)
Hypoxia (Buffa)
d Immune cell infiltration (CRYAB)
Macrophages
Macrophages M2
Macrophages M1
Macrophages M0
Lymphocytes
T cell CD8
T cell CD4 Naive
CD4 Memory resting
CD4 Memory activated

Fig. S4

a

1 MDIAIHHPWIRRPFFPFHSPSRLFDQFFGEHLLESDLFPTSTSLSPFYLRPPSFLRAPSW 60
MDIAIHHPWIRRPFFPFHSPSRLFDQFFGEHLLESDLF T+TSLSPFYLRPPSFLRAPSW
1 MDIAIHHPWIRRPFFPFHSPSRLFDQFFGEHLLESDLFSTATSLSPFYLRPPSFLRAPSW 60

61 FDTGLSEMRLEKDRFSVNLDVKHFSPEELKVKVLGDVIEVHGKHEERQDEHGFISREFHR 120
DTGLSEMRLEKDRFSVNLDVKHFSPEELKVKVLGDVIEVHGKHEERQDEHGFISREFHR
61 IDTGLSEMRLEKDRFSVNLDVKHFSPEELKVKVLGDVIEVHGKHEERQDEHGFISREFHR 120

121 KYRIPADVDPLTITSSLSSDGVLTVNGPRKQVSGPERTIPITREEKPAVTAAPKK 175
KYRIPADVDPLTITSSLSSDGVLTVNGPRKQVSGPERTIPITREEKPAV AAPKK
121 KYRIPADVDPLTITSSLSSDGVLTVNGPRKQVSGPERTIPITREEKPAVAAAPKK 175

b

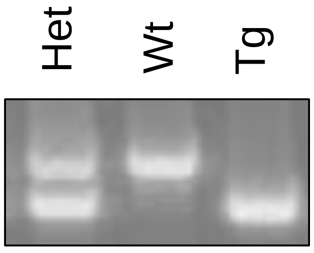

c

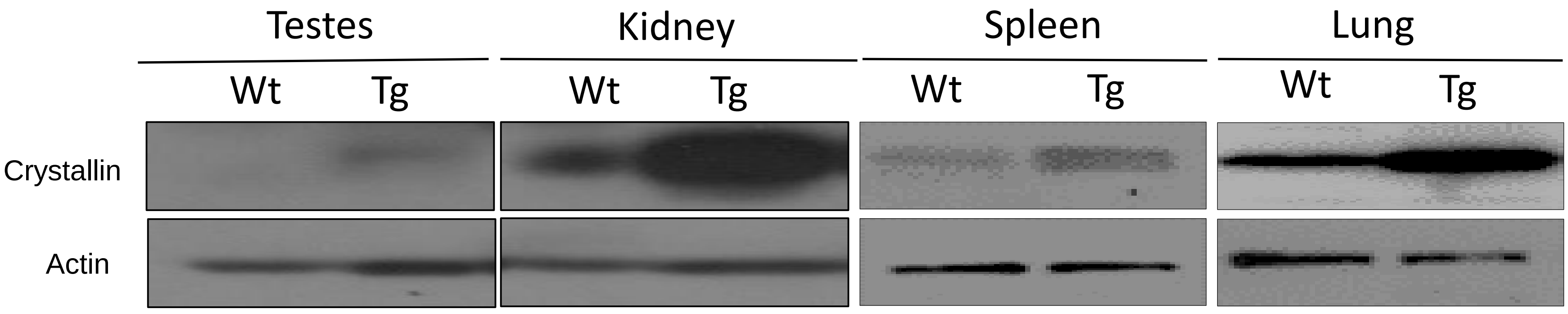

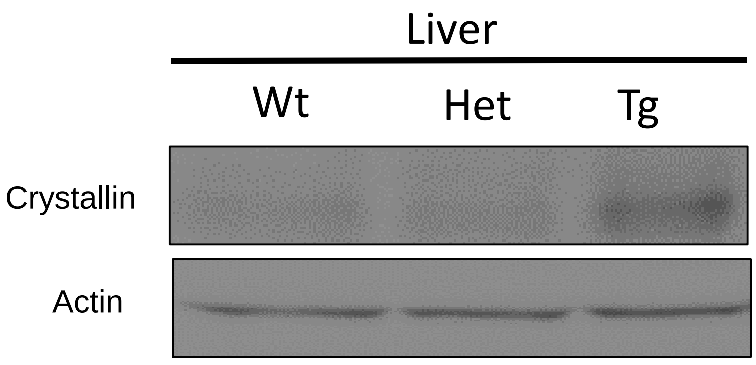

d

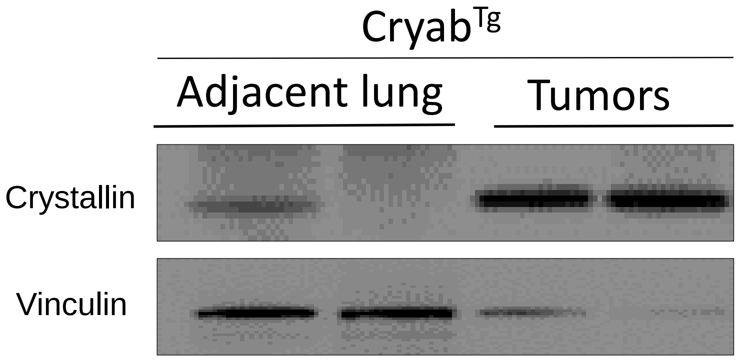

Fig. S5

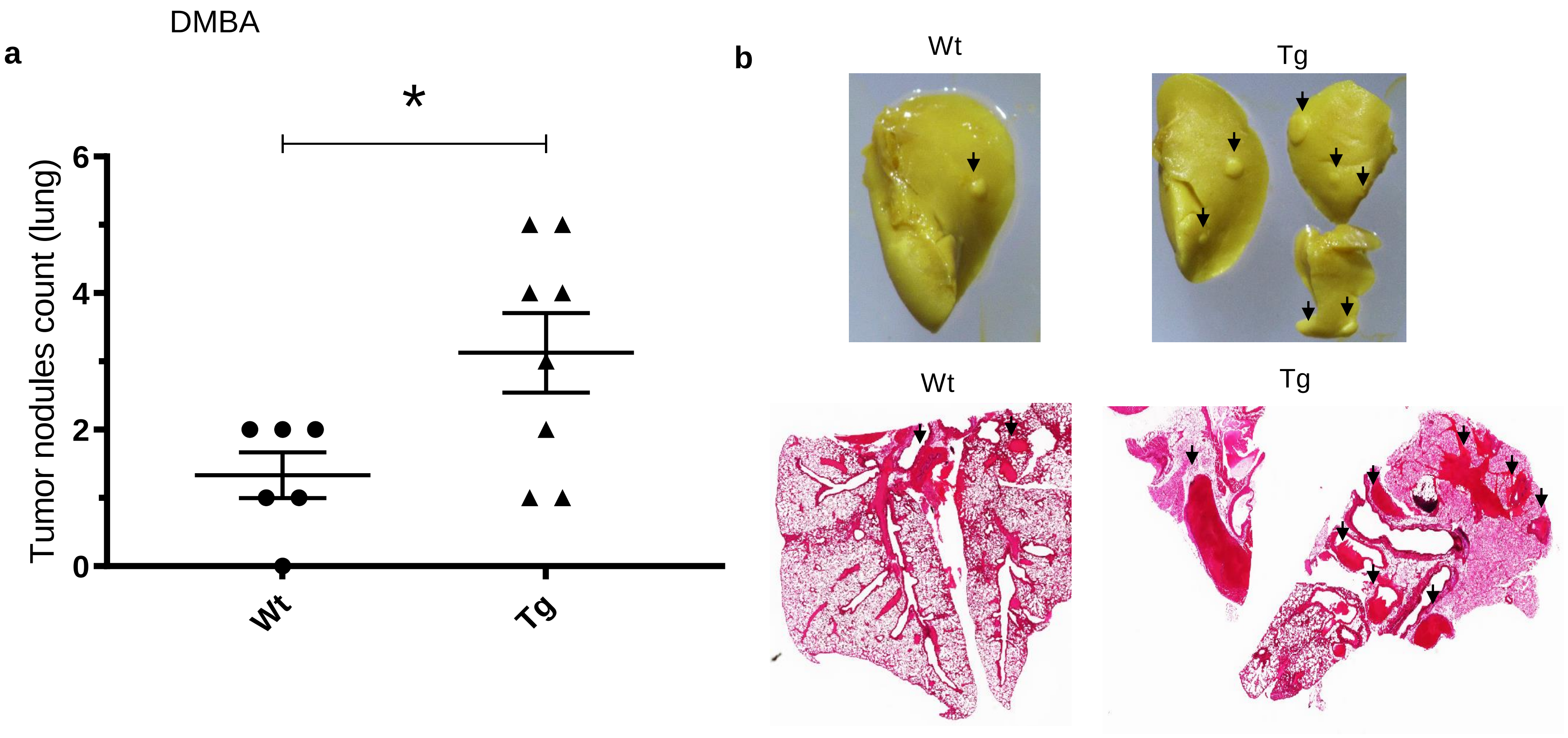

Fig. S6

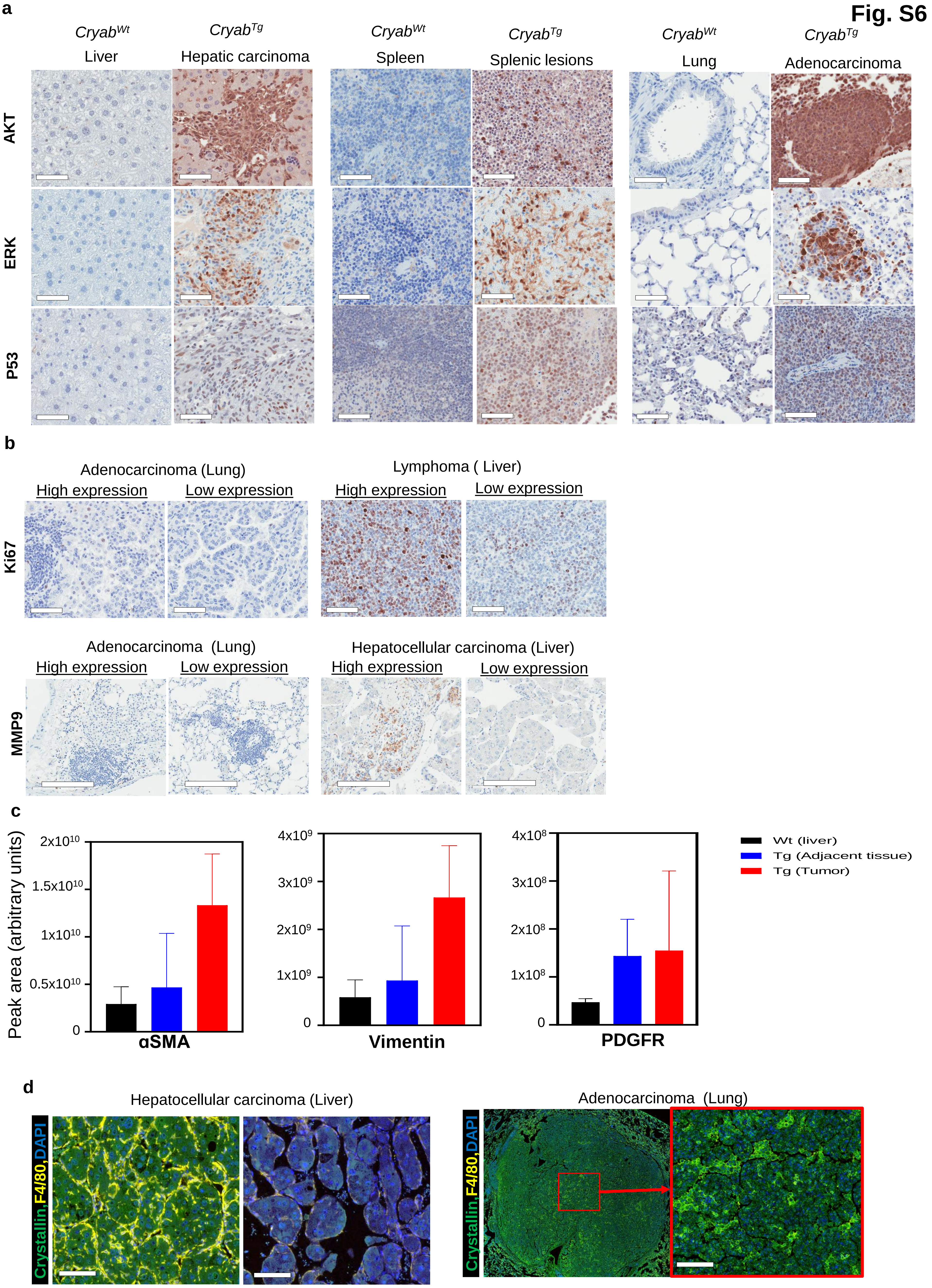
a
CryabWt
Liver
CryabTg
Hepatic carcinoma
CryabWt
Spleen
CryabTg
Splenic lesions
CryabWt
Lung
CryabTg
Adenocarcinoma
AKT
ERK
P53
b
Adenocarcinoma (Lung)
High expression
Low expression
Lymphoma (Liver)
High expression
Low expression
Ki67
Adenocarcinoma (Lung)
High expression
Low expression
Hepatocellular carcinoma (Liver)
High expression
Low expression
MMP9
c
Peak area (arbitrary units)
2x10^10
1.5x10^10
1x10^10
0.5x10^10
0
αSMA
4x10^9
3x10^9
2x10^9
1x10^9
0
Vimentin
4x10^8
3x10^8
2x10^8
1x10^8
0
PDGFR
Wt (liver)
Tg (Adjacent tissue)
Tg (Tumor)
d
Hepatocellular carcinoma (Liver)
Crystallin,F4/80,DAPI
Adenocarcinoma (Lung)
Crystallin,F4/80,DAPI

Fig. S7

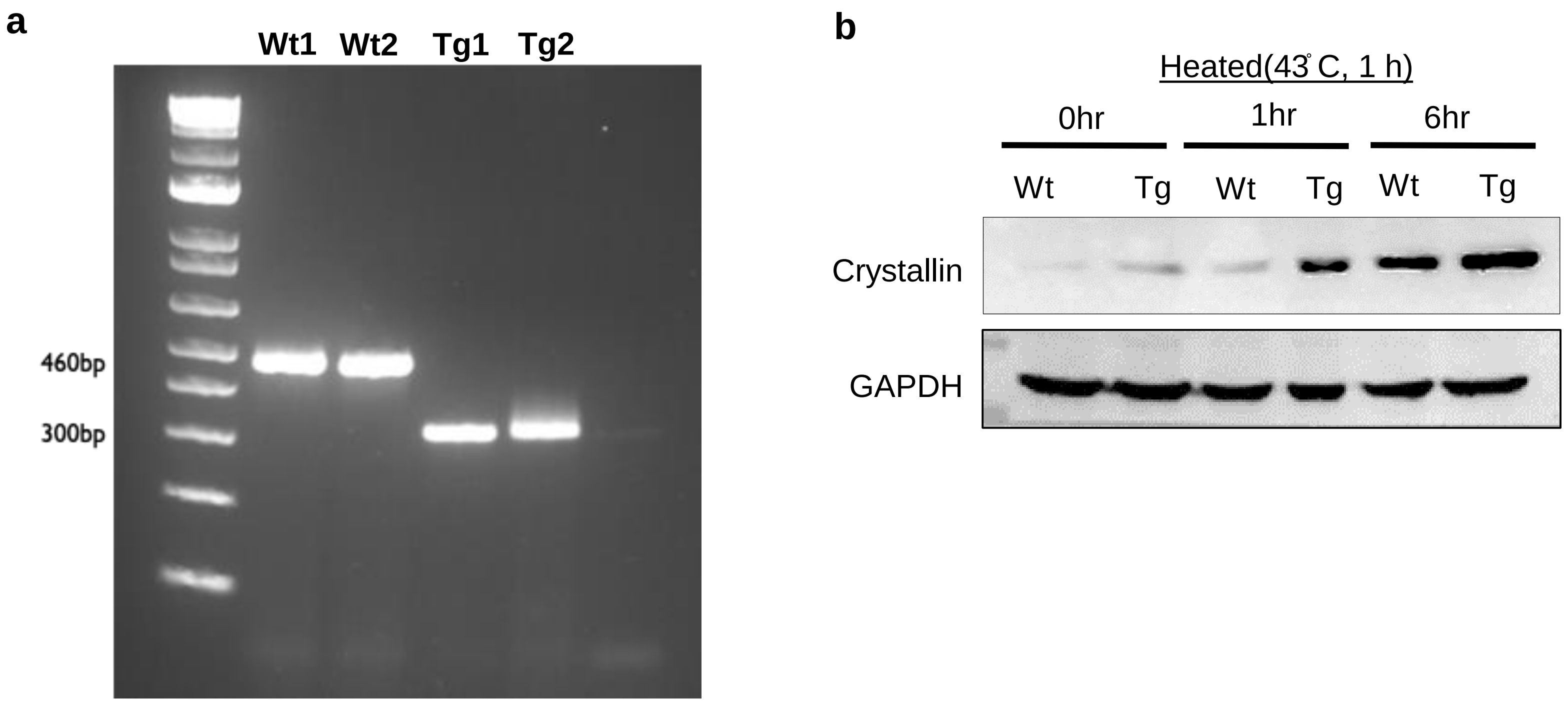
a
Wt1 Wt2 Tg1 Tg2
460bp
300bp
b
Heated(43°C, 1 h)
0hr
1hr
6hr
Wt Tg Wt Tg Wt Tg
Crystallin
GAPDH

a

Fig. S8

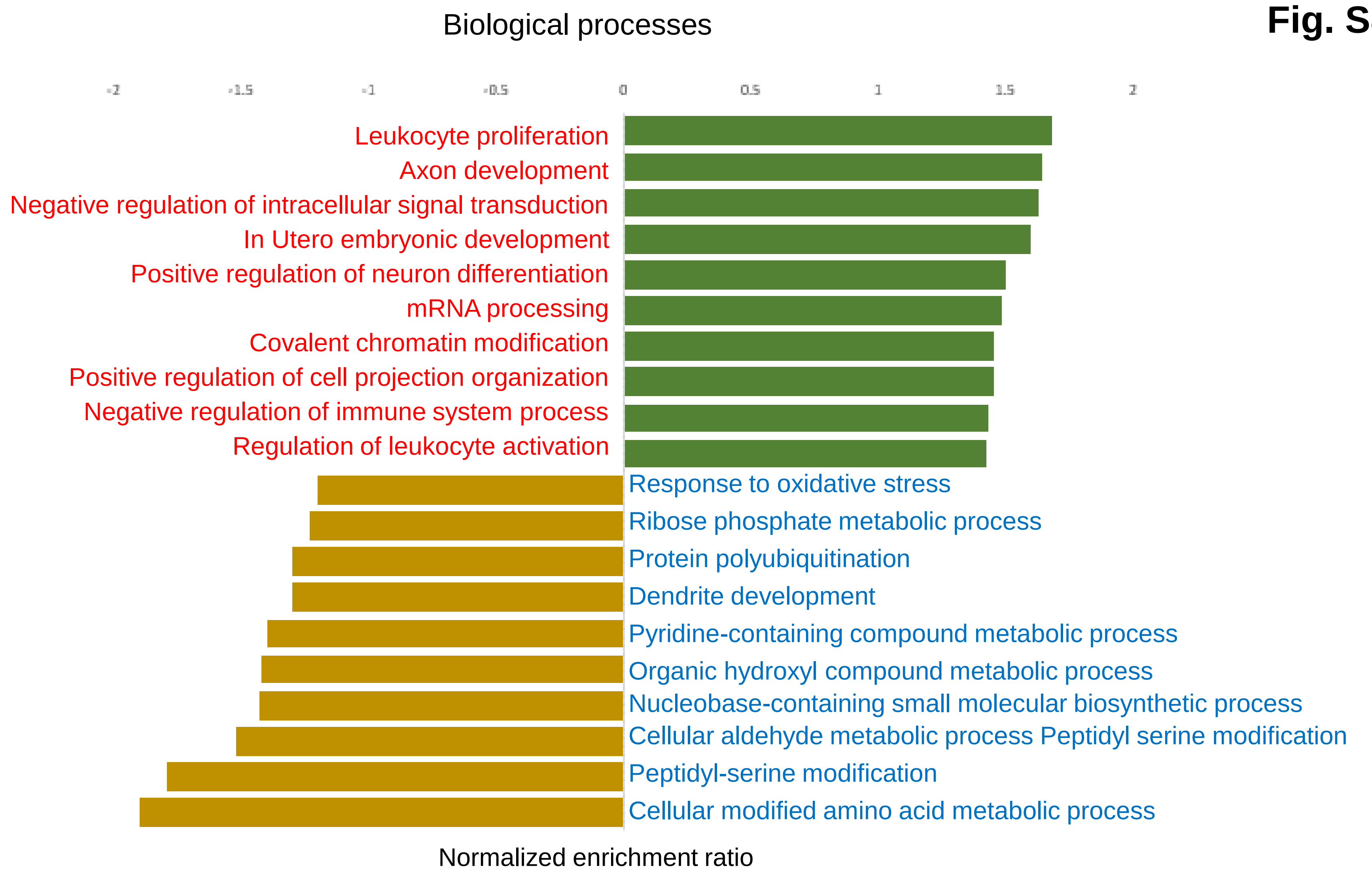

b

**Bsg**	**Basigin**	Extracellular matrix metalloproteinase inducer (EMMPRIN) or (CD147):
Crabp2	Cellular retinoic acid-binding protein 2	Role in metastasis of lung cancer is unclear.
Hells	Lymphocyte-specific helicase	Elevated expression in aggressive metastatic tumor
Sar1b	GTP-binding protein SAR1b	Associated with shorter overall survival time in patients with CRC.
Eif4b	Eukaryotic translation initiation factor 4B	The chemo-resistance and metastasis characteristics of NSCLC

c

**Co-occurrence Analysis**			
**A**	**B**	**p-Value**	**q-Value**
CRYAB	BSG	<0.001	<0.001
CRYAB	HELLS	<0.001	<0.001
BSG	EIF4B	<0.001	<0.001
CRABP2	HELLS	0.005	0.012
HELLS	EIF4B	<0.001	<0.001
HELLS	SAR1B	<0.001	<0.001
SAR1B	EIF4B	0.006	0.012

Fig. S9

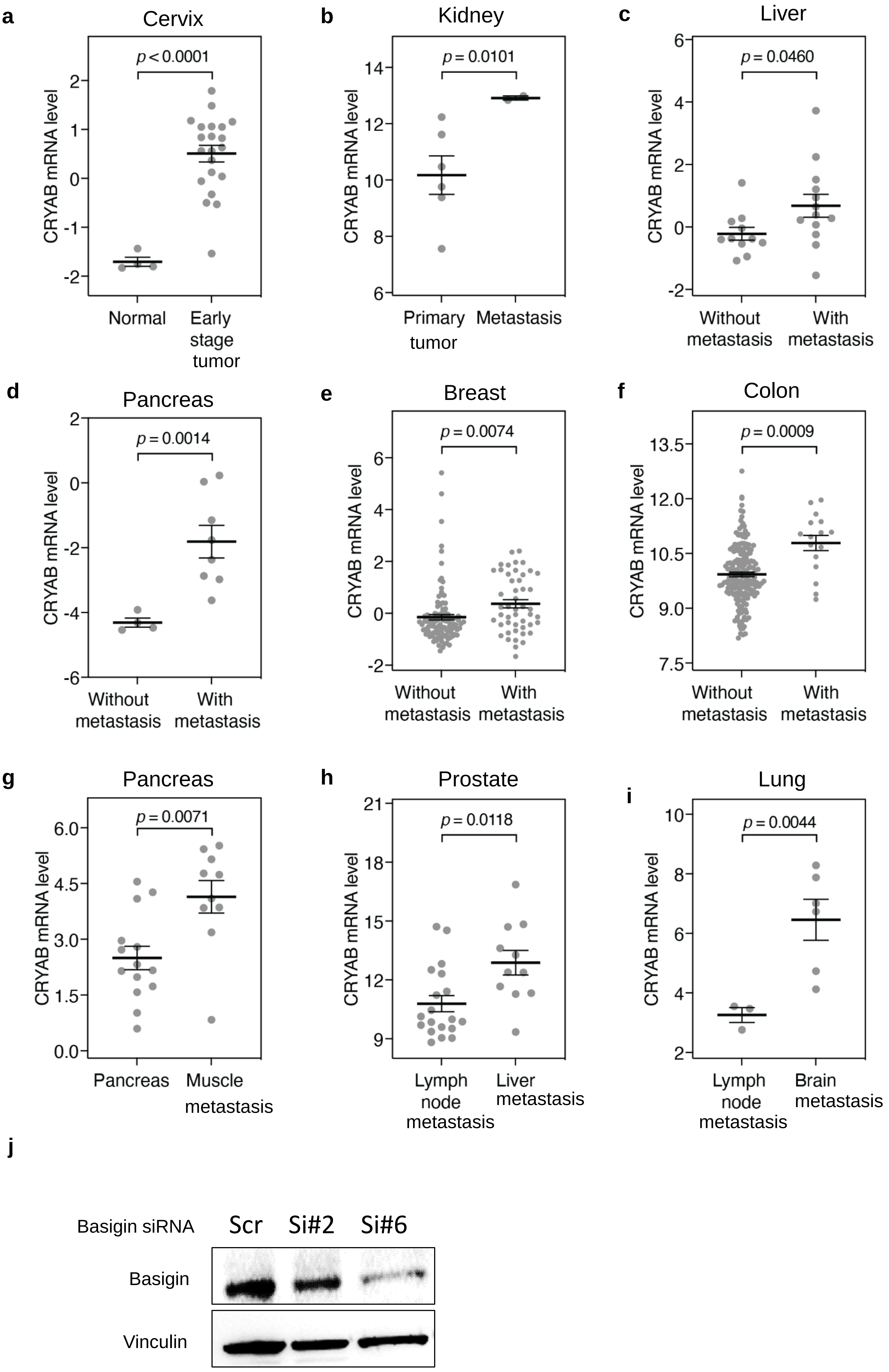
a
Cervix
p < 0.0001
CRYAB mRNA level
2
1
0
-1
-2
Normal
Early stage tumor
b
Kidney
p = 0.0101
CRYAB mRNA level
14
12
10
8
6
Primary tumor
Metastasis
c
Liver
p = 0.0460
CRYAB mRNA level
6
4
2
0
-2
Without metastasis
With metastasis
d
Pancreas
p = 0.0014
CRYAB mRNA level
2
0
-2
-4
-6
Without metastasis
With metastasis
e
Breast
p = 0.0074
CRYAB mRNA level
6
4
2
0
-2
Without metastasis
With metastasis
f
Colon
p = 0.0009
CRYAB mRNA level
13.5
12.0
10.5
9.0
7.5
Without metastasis
With metastasis
g
Pancreas
p = 0.0071
CRYAB mRNA level
6.0
4.5
3.0
1.5
0.0
Pancreas
Muscle metastasis
h
Prostate
p = 0.0118
CRYAB mRNA level
21
18
15
12
9
Lymph node metastasis
Liver metastasis
i
Lung
p = 0.0044
CRYAB mRNA level
10
8
6
4
2
Lymph node metastasis
Brain metastasis
j
Basigin siRNA
Scr
Si#2
Si#6
Basigin
Vinculin
