# Supplementary material for: Alpha-B-Crystallin overexpression is sufficient to promote tumorigenesis and metastasis in mice
Source: Exp Hematol Oncol. 2023 Jan 9;12:4. doi: 10.1186/s40164-022-00365-z (PMC9830749; doi:10.1186/s40164-022-00365-z)
Supplement: Supplementary file 4 — Additional file 4. Supplementary methodology:TCGA data analysis and targeted mass-spectrometry. [file 40164_2022_365_MOESM4_ESM.docx]

**Supplementary methodology:**

**TCGA data analysis**

Signal Transduction Pathways: Pathway scores for each of these eight pathways were determined by integration of multi-platform data from several types of 'omics' analyses, as described by Hoadley et al., 2010 (PMID 25109877).

Hypoxia and angiogenesis: Angiogenesis score: A 43-gene expression signature for the level of angiogenesis, as determined by Masiero et al., 2013 (PMID 23871637). Hypoxia score (Winter): A 99-gene expression signature for the level of hypoxia, as determined by Winter et al., 2007 (PMID 17409455). Hypoxia score (West) : A 26-gene expression signature for the level of hypoxia, as determined by Eustace et al., 2013 (PMID 23820108). Hypoxia score (Sorensen): A 27-gene expression signature for the level of hypoxia, as determined by Sorensen et al., 2010 (PMID 20429727). Hypoxia score (Seigneuric): A gene expression signature for the level of hypoxia, as determined by Seigneuric et al., 2007 (PMID 17532074). Hypoxia score (Ragnum): A 32-gene expression signature for the level of hypoxia, as determined by Ragnum et al., 2015 (PMID 25461803). Hypoxia score (Hu): A 13-gene expression signature for the level of hypoxia, as determined by Hu et al., 2009 (PMID 19291283).

Autophagy and stemness: Autophagy-related prognostic signature: A prognostic gene expression signature based in the weighted expression of 8 autophagy-related genes, as described by An et al., 2018 (PMID 30410611). Stemness (mRNA): Level of stemness, based on mRNA data, determined as described by Malta et al., 2018 (PMID 29625051).

Immune cell infiltration: The levels of indicated tumour-infiltrated immune cell types were estimated using the tumor immune estimation resource, developed by Li et al., 2016 (PMID 27549193).

**Targeted Mass-spectrometry**

In-solution Digestion: Proteins were reduced with 5 mM DTT at 60° C for 30 min and alkylated with 10 mM IAA for 10 min at room temperature in the dark. Proteins were precipitated with 5x ice cold acetone and reconstituted in TEABC, followed by digestion with Trypsin by incubating at 37⁰C overnight. Digestion was quenched by adding trifluoroacetic acid (TFA) to achieve a concentration of 0.5%. Tryptic digests were cleaned using tips packed in-house with Empore SDB-RPS material, dried on a speedvac, and then reconstituted in 0.1% FA for LCMS analysis. Reconstituted peptide concentration was read using nanodrop at 205 nm then adjusted to normalize sample amount loaded for the LC-MS runs.

LC-MS Analysis: Samples were loaded on to a Thermo Acclaim PepMap 100 trap column (5 mm x 300 um ID) for 5 min at a flow rate of 10 ul/min with 95% Solvent A (0.1% FA in water) and subsequently separated on a Thermo PepMap100 analytical column (150 mm x 300 um ID) equipped on a Thermo Ultimate 3000 LC interfaced with Thermo Exactive HF-X mass spectrometer. Peptides were resolved using a linear gradient of 5% solvent B (0.1% FA in 80% ACN) to 40% solvent B over 48 min at a flow rate of 1.5 µl/min. This was followed by column washing and equilibration for a total run time of 65 min. An inclusion list for peptides corresponding to the proteins of interest was generated using previous in-house data acquisition on MEFs as well as publicly available data on the ProteomicsDB website^1^. Parent peptides were detected in the orbitrap at a resolution of 60,000 and those that matched the specifications from the inclusion list were selected for MS2. MS2 was performed using HCD with a collision energy of 30% and an orbitrap resolution of 15,000. Maximum injection time was 27 ms, while the AGC target was 200,000 and the isolation window was 1.2. Data was analysed using Skyline software^2^. Integrations were manually checked and abundance was extracted based on total MS1 peak area.

1. Schmidt, T. et al. ProteomicsDB. Nucleic Acids Res 46, D1271–D1281 (2018).

2. MacLean, B. et al. Skyline: An opensource document editor for creating and analyzing targeted proteomics experiments. Bioinformatics 26, 966–968 (2010).
